# Dancing robots: aesthetic engagement is shaped by stimulus and knowledge cues to human animacy

**DOI:** 10.3389/fnhum.2024.1413066

**Published:** 2024-11-25

**Authors:** Kohinoor M. Darda, Aaron Maiwald, Tanvi Raghuram, Emily S. Cross

**Affiliations:** ^1^Advancement and Research in the Sciences and Arts (ARISA) Foundation, Pune, India; ^2^Institute of Cognitive Science, Universität Osnabrück, Osnabrück, Germany; ^3^Professorship for Social Brain Sciences, ETH Zurich, Zurich, Switzerland

**Keywords:** robots, dance, aesthetics, social robotics, choreography, artificial intelligence

## Abstract

**Introduction:**

Artificial intelligence (AI) and robots are increasingly shaping the aesthetic preferences of art consumers, influencing how they perceive and engage with artistic works. This development raises various questions: do cues to the humanness of the origin of an artwork or artist influence our aesthetic preferences?.

**Methods:**

Across two experiments, we investigated how the perception and appreciation of dance is influenced by cues to human animacy. We manipulated **Agent Form** (human-like or robot-like dancer), **Belief about Movement Source** (human motion capture or computer animation), **Source of Choreography** (human- or computer-generated), and **Belief about Choreography Source** (believed to be human- or computer-generated).

**Results:**

Results pointed toward agent congruence: In **Experiment 1**, robot agents were preferred when the movement source was believed to be computer animation. In **Experiment 2**, robot agents were preferred when the choreography was believed to be computer-generated, while choreographies believed to be human-generated were generally preferred. Participants could not accurately identify the actual source of choreography. These results persisted beyond the effects of age, dance expertise, technological expertise, attitudes toward AI, and perceived familiarity, complexity, evocativeness, technical competence, or reproducibility of the dance. Dance expertise, technological expertise, and attitudes toward AI independently impacted aesthetic judgments.

**Discussion:**

These findings provide insights into the design of robotic dance, highlighting features of dance choreography and audience characteristics that influence aesthetic engagement. To enhance AI-driven creative productions, shaping perceptions will be crucial for better audience reception and engagement.

## Introduction

When The Jackson 5 performed “Dancing Machine” in 1973, the “robot dance” became, for better or for worse, deeply ingrained in dance history.^1^ Variations of this dance remain popular, as numerous YouTube videos and millions of viewers attest. Recently, robots are becoming serious competitors in what is now a kind of mutual imitation game. Instead of human dancers imitating robots, robots and artificial intelligence (AI) are now starting to imitate *and* create dance. Initially used to capture notations and record dance movements in written symbolic forms, the role of AI in dance has evolved considerably over the past decade. Some of AI’s most recent feats in the dance domain include generating sequences of choreography using archives of hundreds of hours long footage of dancers performing (Plone, 2019), as well as impressive videos of robots performing human-like dance from the robotics company “Boston Dynamics,” and choreographies from the “Living Archive,” a tool to create novel dance sequences using AI.

**Figure 1:**
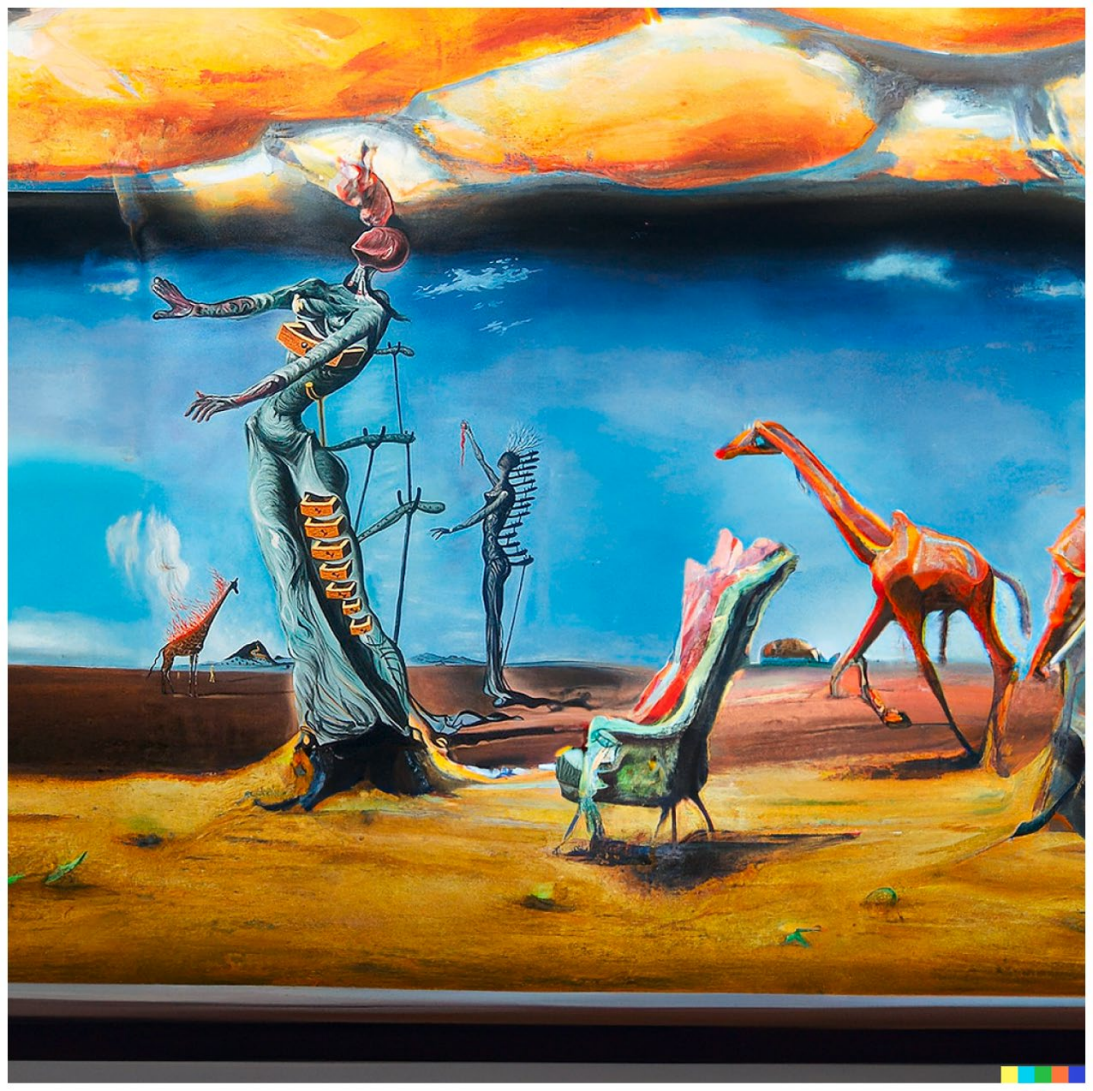
DALL·E 2-generated version of ‘The Burning Giraffe.’ Image generated by OpenAI’s DALL·E 2 using the text prompt ‘A desert populated by burning animals and furniture mannequins in the style of The Burning Giraffe by Salvador Dalí.’ Generated using DALL·E 2 (openai.com) (accessed: 14.09.22).

AI’s development in the world of dance is part of a larger trend of rapid progress in AI development in general.^2^ The subfield working on *artistic* AI is no exception to this. For instance, consider this poem by the English poet Philip Larkin about People: “People/What do people do all day? /Oh, what do people do? /They walk around and around /And then they lie down, /and that is all they do”. Although the plainness and bleakness of this poem are reminiscent of Philip Larkins’ style, he never wrote this poem. It is the creation of a text-based AI, a version of the Generative Pre-trained Transformer 3 (GPT-3), in an attempt to *imitate* Philip Larkin on the topic of people.^3^ GPT-3 is a deep learning model that is trained using internet data to generate any kind of text. This is only one of many impressive examples of recent AI artworks. Figure 1 is an image generated by Open AI’s “DALL: E 2”, an AI that outputs images in response to text prompts (Ramesh et al., 2022).

With this recent progress, public interest in AI-generated artworks is scaling new heights. Google Trends shows that the popularity of the search term “AI art” doubled in the three months between April 2022, when DALL: E 2 was released, and July 2022. In that month, the popularity of the term (as measured by Google) was about four times higher than it had been in the fifteen years prior to 2020.^4^ Not only is there exponential growth in the field of AI art, but there is also a growing acceptance of this coexistence allowing for a new and unique relationship to blossom between AI and human art.

The advancements in technology and AI have forged a special signature for itself in the popular works of choreographers like Wayne McGregor, Garry Stewart, and Bill T. Jones. McGregor especially has chronicled the evolution of AI in many of his well-loved choreographies, representing AI in every extension of dance: the costume, lighting, backdrop project, and sound design to name a few (Preciado-Azanza and Akinleye, 2020). Furthermore, the “Living Archives” project has been instrumental in demonstrating the adaptability of AI in the field of performing arts. This program, is trained on 25 years’ worth of choreographies to predict dance movements in novel combinations. This acts as an extension to the artists’ imagination by going beyond the human mind to create unlimited novel combinations of dance moves. The innovative program is presented as appealing visuals of stick figures that imitate human-like dance movements. While this program initiates conversations surrounding what the future may hold for choreography, it also reorients the interest in dance from performance to creation (Jordan, 2020).

The mass consumption of such artworks is evidence that there is an increasing presence of AI and robots in the realm of art which is moulding the aesthetic identity of the new-age consumer of art. Thus, we see various questions concerning the psychology of aesthetic perception are gaining relevance. These include: do cues to the humanness of the origin of an artwork or artist influence our aesthetic preferences? If so, how do perceptual or *stimulus* cues (e.g., seeing a robot artist) and/or *knowledge* cues (e.g., believing that a painting was made by a human) influence our aesthetic perception?

In the current study, we aim to shed light on the cognitive mechanisms underlying our perception of human and robot dancers, and human- and computer-origins of dance choreography and movement. A convergence of social robotics and cognitive neuroscience evidence suggests that stimulus and knowledge cues to humanness influence social perception and interactions (Stanley et al., 2007; Cross et al., 2016). Stimulus cues include how an agent looks or moves, whereas knowledge cues refer to beliefs about an agent or movement’s origin. But how do these cues to humanness influence our aesthetic preferences over creative works?

Stimulus and knowledge cues to the humanness or human animacy in art have only recently been investigated and are especially relevant in empirical investigations of computer-generated artworks. In the last few years, various studies have demonstrated that people on average prefer and show greater appreciation for human-generated over computer-generated dance, poetry, music and paintings, as well as text-based archives (e.g., Chamberlain et al., 2018; Köbis and Mossink, 2021; Darda and Cross, 2023; Darda et al., 2023). At least in some cases, this preference for human-generated art seems to be caused by inherent features of the artworks themselves (Chamberlain et al., 2018). People tend to prefer human-generated art irrespective of whether they have already consciously judged an artwork as human or computer-generated. However, when they perceive a robot agent making the artwork, their aesthetic appreciation for computer-generated artworks increases, suggesting an influence of the agents’ presence (Chamberlain et al., 2018).

In creative productions such as paintings or poetry, the artist and the artwork are two different entities. That is, when viewing a finished painting, we usually do not see the artist and the finished painting simultaneously within the artwork (although we might see the artist *in process*). Thus, any stimulus cues when perceiving the creative production’s origin (whether human-or computer-generated) are perceived within the painting itself. Compared to paintings or poetry, dance provides a unique use case to investigate stimulus and knowledge cues to human animacy. When watching a dance video, the dancer, the movement, and the dance choreography are all viewed simultaneously. This allows us to investigate at least three distinct components at the same time—the dancing agent, the source of the dance choreography, and the source of the movement seen by the viewers.

Evidence suggests that along with inherent (or bottom-up) stimulus cues about the humanness of artworks, beliefs about the origin of movements result in a preference for movements thought to originate from humans (Cross et al., 2016; Darda and Cross, 2023). For example, Cross et al. (2016) found that actions believed to originate from humans more strongly engage brain regions associated with person perception than actions believed to originate from computers. The same study also reports that people tend to find movements more pleasant and smooth to watch if they believe the movements originated from a human (using human motion capture) rather than from a computer (using computer animation). Likewise, Darda and Cross (2023) showed that people show a bias against dance choreographies they merely believed to be computer-generated even when they were not. In this same study, participants viewed only human-generated dance choreographies, but believed some of these choreographies were computer-generated (anti-computer bias). However, it is unclear whether this bias also persists when participants view computer-generated choreographies but believe some of them are made by a human (pro-human bias).

These findings raise another question: to what extent are people able to consciously distinguish between human and computer artworks? Gangadharbatla (2022) showed very low accuracy for the classification of paintings into AI-art and human-made art. Köbis and Mossink (2020) provide evidence that participants were unable to identify AI-generated poems that were intended to imitate the style of Maya Angelou from human-generated ones. Thus, in some cases, AI already passes a kind of “artistic Turing Test.” These findings also extend beyond the world of art. In experiments that investigated people’s reactions to algorithmically generated news articles or text-based archives and those written by human experts in the field (Clerwall, 2014; Sundar and Nass, 2001; Graefe et al., 2018; Darda et al., 2023), participants were usually unable to distinguish between human- and AI-generated texts in a reliable way.

In contrast, in the context of paintings and music, some studies suggest that participants *are* capable of accurately identifying human and computer creative productions (Chamberlain et al., 2018; Moffat and Kelly, 2006). It is important to note that studies finding people to be largely incapable of accurately distinguishing between computer- and human-generated art are more recent than those who find the opposite. As noted earlier, the field of AI currently progresses at a fast pace^5^ and people’s accuracy in distinguishing human art from computer-generated art plausibly decreases as artistic AI becomes more advanced (assuming that it is trained to imitate human art).

The present study aims to fill crucial gaps in the literature and extend upon the research from Darda and Cross (2023). Here, we investigate how the perception and appreciation of dance are influenced by cues to human animacy by manipulating (1) “agent form”—either a human-like or robot-like dancer; (2) belief about the source of the dance movement—either from human motion capture or computer animation; (3) the source of choreography—either human-or computer-generated; and (4) belief about the source of choreography—whether participants *believe* a choreography is human-or computer-generated. Finally, we also investigate the extent to which participants can recognize the source of choreographies.

In the first experiment, we manipulate agent form and choreography, and belief about the origin of the dance movement to investigate how stimulus and knowledge cues interact to influence aesthetic responses to dance videos. In the second experiment, we investigate whether different responses toward human- and computer-generated creative productions are because of a bias toward humanness or a bias against the artificialness of agent forms and dance choreographies, by manipulating the belief about the source of choreography and agent form. We hypothesize that stimulus and knowledge cues to human animacy will influence aesthetic appreciation. Answers to these questions will have implications across different fields including for artists, specifically dance choreographers, as they decide to what extent they include robotic dancers in their work or whether to use AI tools for the creation of choreographies.

Specifically, for Experiment 1, based on prior research, we predict that choreographies that are human-generated, performed by a human agent, and movements believed to originate from human motion capture would be rated higher on aesthetic variables of beauty, liking, smoothness, how likely they are to watch the video again, and enjoyability, than those performed by a robot agent, computer-generated, and believed to originate from computer animation. We also predict that participants will not be able to accurately identify which choreographies are human- and which are computer-generated accurately.

For Experiment 2, we predict that choreographies believed to be human-generated and performed by a human agent will be rated higher on aesthetic variables compared to those that are believed to be computer-generated. In Experiment 2, we also evaluate whether this bias is found in both conditions: (1) where participants see *only* human-generated choreographies but believe some are computer-generated (even when they are not), and (2) where participants see *only* computer-generated choreographies but believe some are human-generated (even when they are not). We expect a bias toward human-generated choreographies in both conditions.

For both Experiments 1 and 2, we also control for a number of variables that have been known to influence aesthetic ratings in prior work. These include age, dance expertise, attitudes toward AI, ratings of familiarity with the dance choreography, evocativeness, complexity, technical complexity, and difficulty of reproducing the choreography. We further include self-reported expertise with technology. Controlling for these variables allows us to investigate whether stimulus and knowledge cues to human animacy influence aesthetic ratings above and beyond these variables.

## Method

### Open science statement

We detail the methodology for determining the sample size, any exclusions of data, and the comprehensive list of measures employed in the study. The statistical analyses and visualizations of data were done using RStudio. The data analysis plan was preregistered on the Open Science Framework and can be found here (https://osf.io/rxb89/registrations) for both the experiments. The pre-registration for the second experiment can be found here.

For all experiments, mixed effects model analyses were executed using the lme4 package (v.1.1–28) in R v.4.1.2. Post-hoc tests were executed using the emmeans package (v.1.7.2). We used an alpha of 0.05 to make inferences and controlled for multiple comparisons using Tukey-HSD in post-hoc tests.

### Data availability statement

Following open science initiatives, all raw data are available online via the Open Science Framework link (https://osf.io/rxb89) for other researchers to pursue alternative questions of interest.

### Stimuli generation

For both experiments, stimuli were derived as follows: the original set of 32 human- and computer-generated choreographies (16 each) were taken from Darda and Cross (2023). A professional Bharatanatyam dancer learnt the choreographies and shot them in front of a green screen. The style of dance chosen was Bharatanatyam, a classical Indian dance form that combines storytelling with abstract and technical dance movements. In addition to the first author having extensive expertise in the dance form, the rationale for choosing Bharatanatyam is that it has a vast and unique vocabulary consisting of hand gestures as well as nuanced leg, torso, and eye movements. Any combination of individual Bharatanatyam movements will yield a coherent and sensible visual. Therefore, when randomly generating sequence of movements for the “computer-generated” choreographies, the resulting choreographies would not violate any rules of choreography and would yield visually coherent and sensible dance sequences.

Each of the human-generated dance choreographies consisted of 8 movement sequences derived from Bharatnatyam choreographies which only used technical and abstract movements and did not involve any interpretive enactments or storytelling. Each of the 8 individual movements or “steps” was extracted from the human-generated choreographies (16×8 = 128) and then labelled chronologically to be used in randomly generating 8 numbers. The movements corresponding to each of these numbers were combined to form a new sequence of choreographies. These movements were then recorded by a dancer, thus creating videos for the computer-generated choreographies.

Initial video editing was done on iMovie where the videos were cut to about 10–11 s duration. Next, each clip was uploaded to DeepMotion Animate 3D software to animate the videos. Characteristics were defined for a robot and human avatar – robot characteristics were as default in the software, and human characteristics were automatically chosen by the software depending on the dancer’s characteristics. The video was presented in grayscale, with a grey background, and a minimalist grid patterned floor to give a 3D effect. The last one second of the video faded out (after the dancer had finished moving).

## Experiment 1

### Participants

The sample size we aimed to recruit was 80 participants (or 80 usable data sets). The sample size was based on power analysis of pilot data, and was performed using the package *simr* (Green and MacLeod, 2016). We found that with *N* = 60 we have >80% power and with *N* = 80 we have >90% to detect our effect of interest, i.e., a three-way interaction between agent, choreography, and belief about the source of movement. We received 108 responses for the first experiment, but 23 participants did not pass the attention test and 22 participants did not understand task instructions. Participants who were 3SD away from the mean duration (Mean = 43.975 min SD = 15.03 min) taken to complete the experiment were excluded (one participant was excluded). The final sample size consisted of 62 participants (33 men, 27 women, and 2 non-binary people; Mean _age_ = 33.40, SD_age_ = 10.30; see Supplementary Table S1 for sample demographics) with complete responses. A linear mixed effect model with agent, choreography, and belief about source of movement as fixed effects was used for the first experiment’s data analysis. The participants were recruited using Prolific and were paid 6GBP/h. Each experiment lasted for approximately 60 min. We excluded individuals who previously participated in a similar study conducted by the lab. Informed consent was taken before participants began the study.

### Procedure

#### Video presentation

The study started with explaining to the participants how movement can be sourced with either human motion capture or with computer animation. Videos were used to demonstrate these processes.^6^ Participants were then asked questions about these videos to ensure they understood what the videos meant, and were clear on how computer animation videos and human motion capture videos were created.

**Figure 2:**
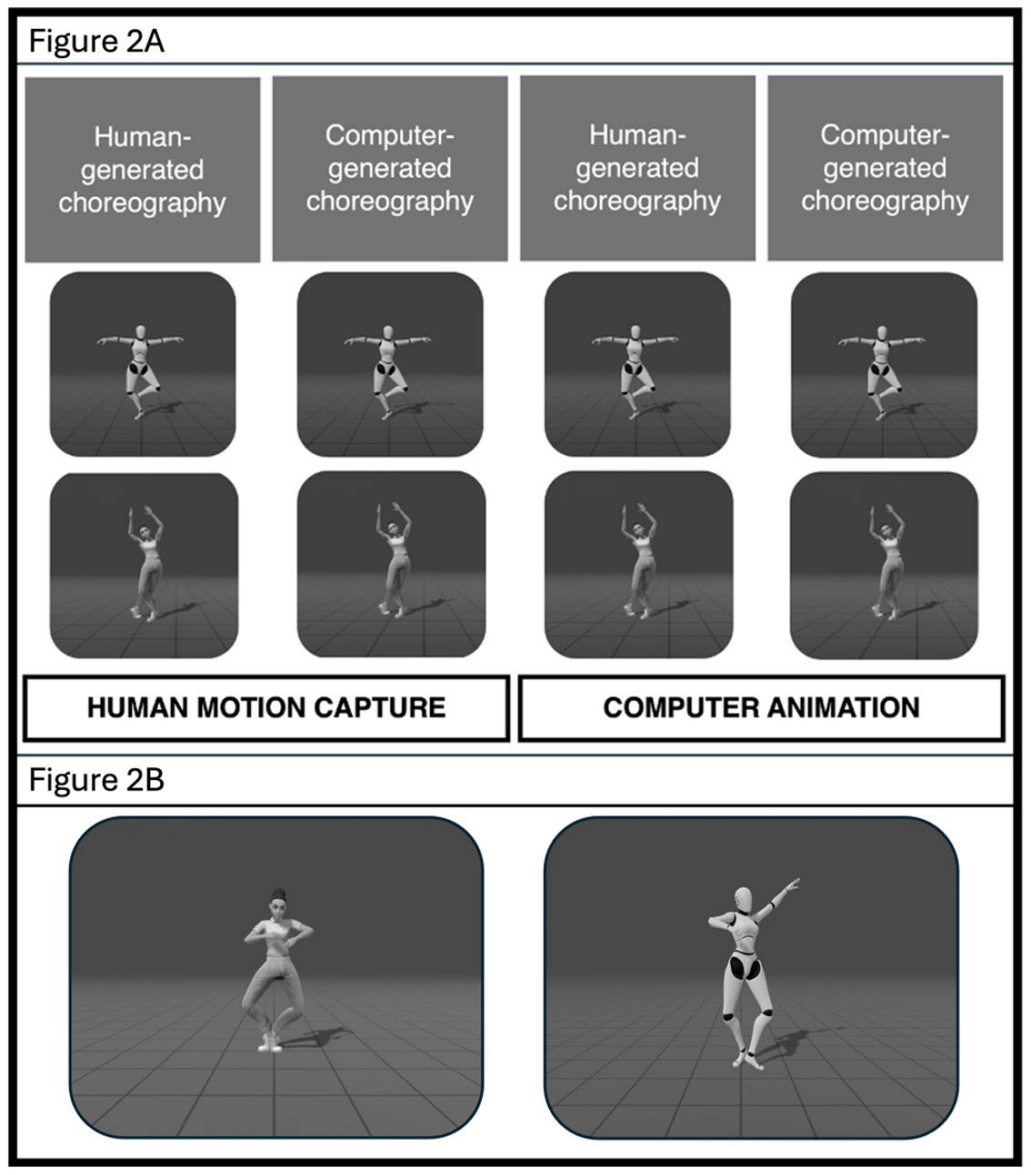
**(A)** Exp 1. 2D representation of the Stimuli used in the current experiments. Human (bottom row) and robot avatars (top row; agent) performed human- and computer-generated choreographies (source of choreography). Source of movement was manipulated to make participants believe that approximately half of the movements are made using computer animation, and half of the movements are made using human motion capture. **(B)** Exp 1. This figure gives a closer look at the stimuli background and the avatars.

The stimulus set included 16 videos, with 8 human-generated and 8 computer-generated choreographies. All 16 choreographies were performed both by a human and a robot agent. Thus, the participants were shown 32 videos in two blocks of 16 videos each consisting of four human-generated choreographies and four computer-generated choreographies each performed by a human and a robot agent. The source of choreography as being human-generated or computer-generated was not revealed to the participants. The first block was labelled as “human motion capture” and the second was labelled “computer animation”. The order of presentation of the videos, as well as which videos were labelled as human motion capture, and which were labelled as computer animation was counter-balanced across all participants. The individual videos were presented in a random order across participants. Figure 2A Exp 1 and Figure 2B Exp 1 show the stimuli used in the current experiment.

#### Rating and categorization task

The participants were asked to rate each video on a scale of 1 (*not at all*)—100 (*extremely*) for the following dependent variables: smoothness, liking, beauty, enjoyability, and whether they would like to see the video again (“watch-again”). The participants were also asked to rate on a similar scale for control variables of “familiarity”, “complexity”, “difficulty”, “evocativeness”, and “technical competency”. Once they completed the rating task, they were presented with all the videos again and asked to categorize them as either computer generated or human-generated choreographies. They also answered demographic questions, questions about their dance and technological expertise, and the General Attitudes towards Artificial Intelligence Scale (GAAIS; Schepman and Rodway, 2020). For questions asked to measure expertise with technology and dance, please see the Supplementary material.

### Data analysis

Responses for attitudes towards AI were calculated and segregated into negative (dystopian) and positive scales (utilitarian; Schepman and Rodway, 2020). Higher scores on both scales suggest positive attitudes toward AI. Both scales had good internal reliability with alpha values 0.74 and 0.87, respectively. Accuracy for categorization of source of choreography was measured and a paired t-test was conducted to compare accuracy between the responses for human-generated choreography and computer-generated choreography.

While we used a mixed-effect maximal model to measure a three-way interaction of agent, source of choreography, and source of movement, we reduced the model complexity because the maximal model did not converge. The model used (for each dependent variable (DV) separately) included the interaction between agent, source of choreography, and belief about source of movement as fixed effects and by-participant and by-item random effects. Table 1 reports all the models for Experiments 1 and 2.

**Table 1:** Model information for Experiments 1 and 2.

Experiment	Dependent variables	Model	Fixed effects	Random effects
1	Beauty, liking, smoothness, enjoyability, watch again	Simple	Interaction between Agent, Source of choreography, and Belief about source of movement	Subject ID, Item
1	Beauty, liking, smoothness, enjoyability, watch again	Full	Same as simple model + familiarity, complexity, evocativeness, difficulty, technical competency, age, attitudes toward AI, expertise with dance, expertise with technology	Subject ID, Item
2	Beauty, liking, smoothness, enjoyability, watch again	Simple	Interaction between Agent, Belief about source of choreography, and group (all CG or all HG)	Subject ID, Item
2	Beauty, liking, smoothness, enjoyability, watch again	Full	Same as simple model + familiarity, complexity, evocativeness, difficulty, technical competency, age, attitudes toward AI, expertise with dance, expertise with technology	Subject ID, Item

The dependent variables were measured on a scale of 1 to 100 with 1 corresponding to “not at all” and 100 corresponding to “extremely.” To further assess if the interaction prevailed when accounting for control variables the model was modified to accommodate “Familiarity”, “Complexity”, “Evocativeness”, “Difficulty”, and “Technical Competency” as fixed effects. While these were the control variables we pre-registered, we further included the control variables of age, attitudes toward AI, expertise with dance, and expertise with technology as fixed effects in the model. The pre-registered model, and the model with additional variables showed comparable results. Therefore, we report only the model with all variables in the main text of the paper.

In the post-hoc analyses, estimated marginal means were calculated for the significant interaction in our main models. We further ran same three-way models again while including the choreography as categorized by the participants. Finally, we also considered whether participants believed there was any manipulation involved when the choreographies were shown and ran the analyses again with only those participants who believed in our manipulation. We found that in both cases (i.e., when source of choreography was categorized by participants, as well as when only participants who fell for our manipulation about the source of movement were included) results were similar to when we included all participants and used the original source of choreography. Therefore, we present results only from all participants and with the original source of choreography in the main text of the paper.

### Results

#### Accuracy

To address the research question of whether the participants would be able to accurately categorize the source of choreography, summary statistics for this sample (*n* = 62) were calculated (see Table 2). To further test this, we first conducted a one sample t-test to check for each source of choreography and found that although the mean for each group was slightly above the hypothesized value (true mean = 0.5) the data is not strong enough to make a definitive conclusion (t_hg_ = 0.64 *p_hg_* = 0.261; t_cg_ = 0.86 *p_cg_ =* 0.464). Additionally, we conducted a paired sample t-test for accuracy in categorization between human and computer-generated choreographies (*t* = −0.37 *p* = 0.709) that revealed no statistically significant difference between the mean accuracy for both sources of choreographies.

**Table 2:** Accuracy of categorization of source of choreography.

Source of- choreography	Agent	Mean accuracy	Standard deviation
Computer generated	Robot	0.48	0.25
Computer generated	Human	0.52	0.25
Human generated	Robot	0.55	0.23
Human generated	Human	0.48	0.26

#### Aesthetic ratings

##### Beauty

**Figure 3:**
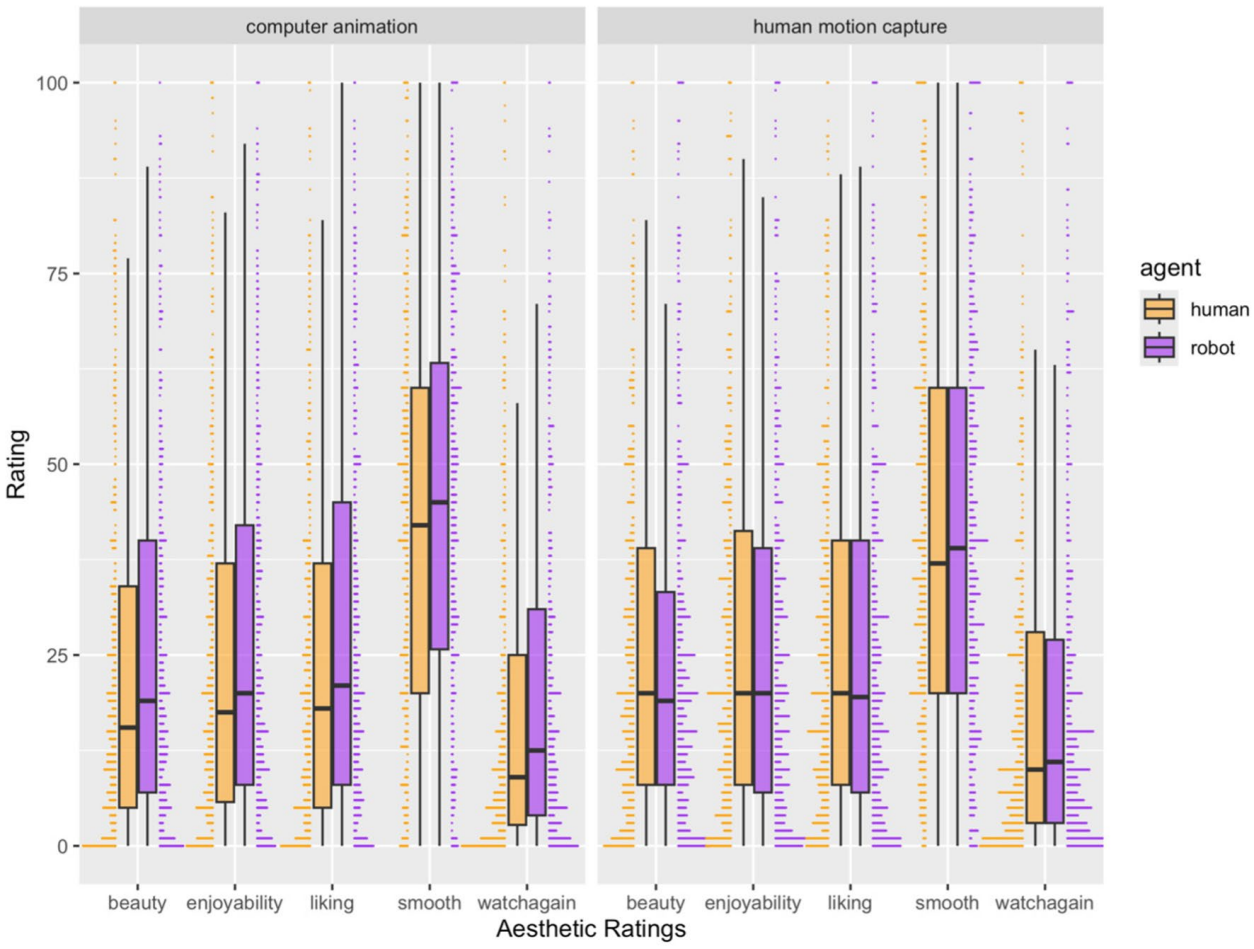
Exp 1. Aesthetic ratings for agent (robot, human) and belief about source of movement (computer animation, human motion capture), collapsed across source of choreography. Only beauty ratings were predicted by a significant interaction effect between the agent and the source of movement.

For the model including only the three-way interaction between source of choreography, agent, and belief about the source of movement (*simple model*), only the interaction between agent and belief about source of movement predicted beauty ratings (*β* = 3.41, *p* = 0.067, 95% CI [−0.23, 7.04]; see Figure 3 Exp 1). No other main effects or interactions were significant. Post hoc tests suggested that robot agents were rated as more beautiful than human agents when the source of movement was believed to be computer animation (estimate = 2.25, SE = 1.48, *p* = 0.136, 95% CI [−0.74, 5.25]), and human agents were rated as more beautiful than robot agents when the source of movement was believed to be human motion capture (estimate = −1.15, SE =1.50, *p* = 0.448, 95%CI [−4.19, 1.88]), although pairwise comparisons were not significant at our statistical threshold.

However, this interaction did not hold when we accounted for control variables. For the model including all control variables (*full model*), results suggested that the two-way interaction between source of choreography and belief about the source of movement predicted ratings of beauty (*β* = 2.31, *p* = 0.035, 95% CI [0.16, 4.46]), and the main effect of belief about source of movement predicted ratings of beauty (*β* = 2.31, *p* = 0.035, 95% CI [0.16, 4.46]). As expected, familiarity, complexity, evocativeness, and technical competency all predicted ratings of beauty. No other main effects or interactions were significant (see Supplementary Tables S2, S3).

Post-hoc results suggested that when the source of movement was believed to be computer animation, computer-generated choreographies were rated as more beautiful than human-generated choreographies, and when the source of movement was believed to be human motion capture, human-generated choreographies were rated as more beautiful than computer-generated choreographies (*p* = 0.035), although pairwise comparisons were not significant at our statistical threshold (ps > 0.05).

##### Liking

For the simple model, the three-way interaction between agent, source of choreography, and belief about source of movement (*β* = 9.11, *p* = 0.009, 95% CI [−2.30, 15.93]), as well as the two-way interaction between agent and belief about source of movement (*β* = 5.66, *p* = 0.001, 95% CI [2.25, 9.07]) predicted ratings of liking. No other main effects or interactions were statistically significant. To further explore the three-way interaction, we tested a two-way interaction between agent and source of choreography separately for movement believed to originate from computer animation, and movement believed to originate from human motion capture. *Post hoc* tests suggested that a two-way interaction between agent and source of choreography significantly predicted liking ratings only when the source of movement was believed to be computer animation (*p* = 0.015) and not when it was believed to be human motion capture (*p* = 0.18). When the source of movement was believed to be computer animation, computer-generated choreography was liked more than human-generated choreography, when performed by a human agent (*p* = 0.029) but not when performed by a robot agent (*p* = 0.486).

However, this three-way interaction did not hold when control variables were accounted for in the model. For the full model including all control variables, familiarity, complexity, technical competency, and evocativeness positively predicted ratings of liking (all ps < 0.001; see Supplementary Tables S4–S7). The interaction between agent and belief about source of movement marginally predicted liking (*β* = 1.84, *p* = 0.067, 95% CI [−0.13, 3.81]). *Post hoc* tests suggested that robot agents were liked more than human agents when the source of movement was believed to be computer animation (estimate = 1.690, SE = 0.705, 95% CI [0.31, 3.07], *p* = 0.017). Human agents were liked more than computer agents when the source of movement was believed to be human motion capture, although this difference was not statistically significant (estimate = −0.15, SE = 0.71, 95% CI [−1.55, 1.25], *p* = 0.829; see Figure 3 Exp 1).

##### Smoothness

For the simple model, no main effects or interactions were statistically significant. The full model including all variables found that familiarity, complexity, evocativeness, and technical competency positively predicted smoothness ratings (all ps < 0.001). Belief about the belief about source of movement marginally predicted ratings of smoothness (*β* = −1.48, *p* = 0.068, 95% CI [−3.08, 0.11]) with videos believed to be created from computer animation rated as smoother than those from human motion capture. No other main effects or interactions were significant (see Supplementary Tables S8, S9).

##### Watch again

For the dependent variable of “watch again” i.e., how much participants wanted to watch the video again, the simple model showed that the two-way interaction between agent and source of movement (*β* = 3.47, *p* = 0.022, 95% CI [0.50, 6.44]) and the main effect of agent (*β* = −1.88, *p* = 0.014, 95% CI [−3.37, −0.38]) significantly predicted ratings of “watch again.” *Post hoc* tests suggested that the robot agent was rated higher on how likely people were to watch the video again compared to the human agent, but only when the source of movement was believed to be computer animation (estimate = 3.615, SE = 1.21, *p* = 0.005, 95% CI [1.18, 6.05]), but not when the source of movement was believed to be human motion capture (estimate = 0.14, SE = 1.23, *p* = 0.907, 95% CI [−2.33, 2.62]).

However, this two-way interaction did not persist when controlling for other variables. The full model with all variables included showed that familiarity, complexity, evocativeness (ps < 0.001) and technical competency (*p* = 0.003) positively predicted ratings of “watch again.” A main effect of agent was also found such that robot avatars were rated higher on the “watch again” variable compared to human avatars (*β* = −1.33, *p* = 0.009, 95% CI [−2.33, −0.33]; see Supplementary Tables S10, S11).

##### Enjoyability

For the simple model, a two-way interaction between agent and belief about the source of movement predicted ratings of enjoyability (*β* = 5.16, *p* = 0.008, 95% CI [1.34, 8.98]). *Post hoc* tests suggested that a robot agent was found to be more enjoyable than a human agent but only when the source of movement was believed to be computer animation (estimate = 3.43, SE = 1.56, *p* = 0.033, 95% CI [0.29, 6.57]). The human agent was rated as more enjoyable than the robot agent but only when the source of movement was believed to be human motion capture, although this difference was not statistically significant (estimate = −1.73, SE = 1.58, *p* = 0.280, 95% CI [−4.91, 1.46]; see Figure 3 Exp 1).

This interaction, however, did not persist in the full model. Only familiarity, evocativeness, complexity, and technical competency (all ps < 0.001) positively predicted ratings of enjoyability. No other main effects or interactions were significant (see Supplementary Tables S12, S13).

## Experiment 2

### Participants

The second experiment included the three-way interaction between agent, the belief was about the source of choreography being either human generated or computer generated, and actual choreographies (whether all choreographies presented were computer-generated or human-generated) as fixed effects. Like Experiment 1, with *N* = 60 we have >80% power and with *N* = 80 we have >90% to detect our effect of interest (given pilot data).

For the "all CG” group (see Procedure), 89 participants started the survey, of which 78 completed it. Seven participants did not understand task instructions, four participants failed the attention check questions, three participants were excluded because of missing data, twelve participants were excluded for being 3 standard deviations away from the mean time taken to complete the experiment, and 17 participants were excluded as they reported that they did not fall for our manipulation. Thus, the final sample for the “all CG” group consisted of 35 participants (17 men, 17 women, 1 non-binary person, Mean_age_ = 39.82, SD_age_ = 13.89).

For the “all HG” group, 87 participants started the survey, of which 79 completed it. Five participants did not understand task instructions, one participants failed the attention check questions, twelve participants were excluded for being 3 standard deviations away from the mean time taken to complete the experiment, two participants were excluded as they reported that they did not fall for our manipulation, and a further two participants were excluded due to missing data. Thus, the final sample for the “all HG” group consisted of 57 participants (27 men, 30 women, Mean_age_ = 36.47, SD_age_ = 11.69).

Like Experiment 1, the participants were recruited using Prolific and were paid 6GBP/h. Each experiment lasted for approximately 60-min. We excluded individuals who previously participated in a similar study conducted by the lab. Informed consent was taken before participants began the study.

### Procedure

Participants were informed that they would see human- and computer-generated dance choreographies. A video explaining how computer-generated choreographies were created was shown to participants, followed by a question to gauge whether participants understood the video (video uploaded on the OSF link: https://osf.io/rxb89/). Sixteen videos were shown to participants – approximately half of the participants saw all 16 computer-generated choreographies (all CG group), whereas the remaining participants saw all 16 human-generated choreographies (all HG group). We manipulated participants’ belief such that the “all CG” group of participants believed that approximately half of the computer-generated choreographies they saw were human-generated. And the “all HG” group pf participants believed that approximately half of the human-generated choreographies they saw were computer-generated. Thus, if a bias against computer-generated choreography is found in the “all CG” group, one explanation for that bias is that participants show a *pro-human* bias, whereas if a bias is found in the “all HG” group, participants may show an anti-computer bias.

For both groups of participants, the 16 videos included 8 choreographies that were presented twice – once by a human agent, and once by a robot agent. The videos were presented in two blocks, one preceded by “all choreographies that you will now see are human-generated choreographies” and the other as “all choreographies that you will now see are computer-generated.” The order of the videos and the blocks was randomized across participants. The participants were not aware that within each block they would see performances by human and robot agents.

#### Rating task

The rating task was the same as Experiment 1.

### Data analysis

Positive and negative scales of the attitudes toward AI scale (GAAIS) were calculated for the final sample for the “all CG” group (*n* = 35) which had good internal reliability with alpha values 0.85 and 0.84, respectively. Sample demographics are reported in Supplementary Table S14. The positive and negative scales of the attitudes toward AI scale (GAAIS) for the “all HG” group (*n* = 57) were calculated which also had good internal reliability with alpha values 0.89 and 0.88, respectively.

Similar to Experiment 1, we used linear mixed models to investigate whether agent (human, robot) and belief about source of choreography (human-generated, computer-generated) influenced aesthetic ratings for participants in both “all CG” and “all HG” groups, i.e., participants who saw only all original computer-generated choreographies (and believed some were human-generated), and participants who saw only all original human-generated choreographies (and believed some were computer-generated). While we pre-registered a two-way interaction separately for these two groups, we collapsed data across the two groups, and included a three-way interaction between agent (robot, human), belief about source of choreography (computer-generated, human-generated), and group (all CG, all HG) as a fixed effect in the model to be able to compare between the two groups. Separate analyses showed comparable results. Similar to Experiment 1, we simplified the model structure as models did not converge with the maximal structure.

The simple model used included agent, belief about source of choreography and group (whether participants saw all choreographies that were actually human-generated, or all choreographies that were actually computer-generated; all HG or all CG group) as fixed effects and by-subject and by-item random effects. Like Experiment 1, we also included control variables in the full model to see if our effects of interest persisted above and beyond the influence of control variables.

### Results

#### Aesthetic ratings

##### Beauty

**Figure 4:**
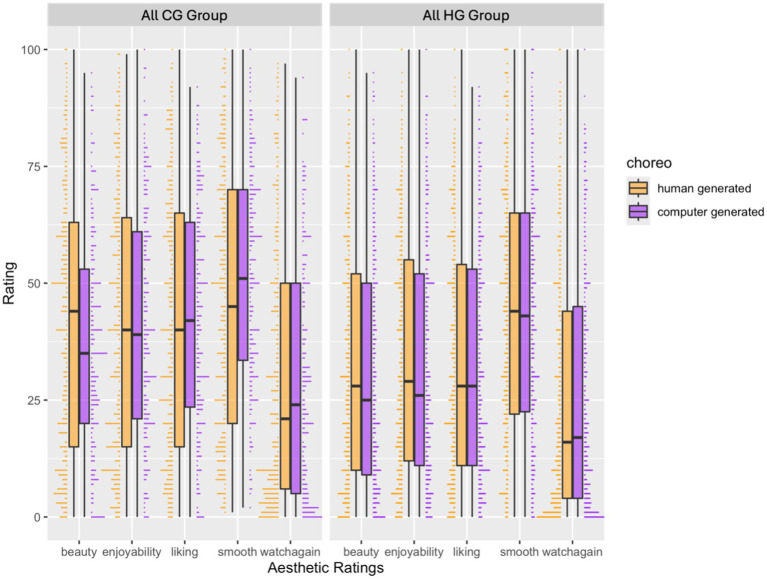
Exp 2. Interaction between belief about source of choreography (human-generated, computer-generated) and group (all CG, all HG) for all ratings. NB: choreo = belief about source of choreography for Experiment 2. Choreographies believed to be computer generated received lower beauty ratings compared to choreographies believed to be human generated.

For the simple model including the three-way interaction between belief about the source of choreography, agent, and group (i.e., participants who saw only all original computer generated choreographies, or participants who saw only all original human generated choreographies), the interaction between belief about the source of choreography and group (*β* = −9.05, *p* = 0.001, 95% CI [−14.61, −3.49]), and the main effect of agent (*p* = 0.048), belief about source of choreography (*p* < 0.001), and group (*p* < 0.001) predicted beauty ratings. *Post hoc* tests suggested that choreographies believed to be computer-generated were rated as less beautiful than choreographies believed to be human-generated, more so in the “all CG” group that saw all original computer-generated choreographies (*p* < 0.001), compared to the “all HG” group (*p* = 0.010) that saw all original human-generated choreographies (*p* < 0.001; see Figure 4 Exp 2).

However, this interaction was only marginally significant in the full model where we added all our control variables. For the full model, familiarity, complexity, evocativeness, and technical complexity positively predicted beauty ratings (all ps < 0.001), difficulty marginally negatively predicted beauty ratings (*p* = 0.076), and self-reported experience with technology negatively predicted beauty ratings (*p* < 0.001). The main effects of agent (*p* = 0.004) and group (*p* < 0.001) predicted beauty ratings, with higher ratings for robot agents and higher ratings for the “all CG” group, and the main effect of belief about source of choreography predicted beauty ratings (*p* = 0.025) with higher ratings for choreographies believed to be human-generated than computer-generated. The two-way interaction between agent and group significantly predicted beauty ratings (*β* = 3.87, *p* = 0.011, 95% [CI 0.88, 6.87]). The two-way interaction between belief about source of choreography and group was only marginally significant (β = −3.22, *p* = 0.081, 95% [CI −6.84, 0.40]; see Supplementary Tables S15, S16).

**Figure 5:**
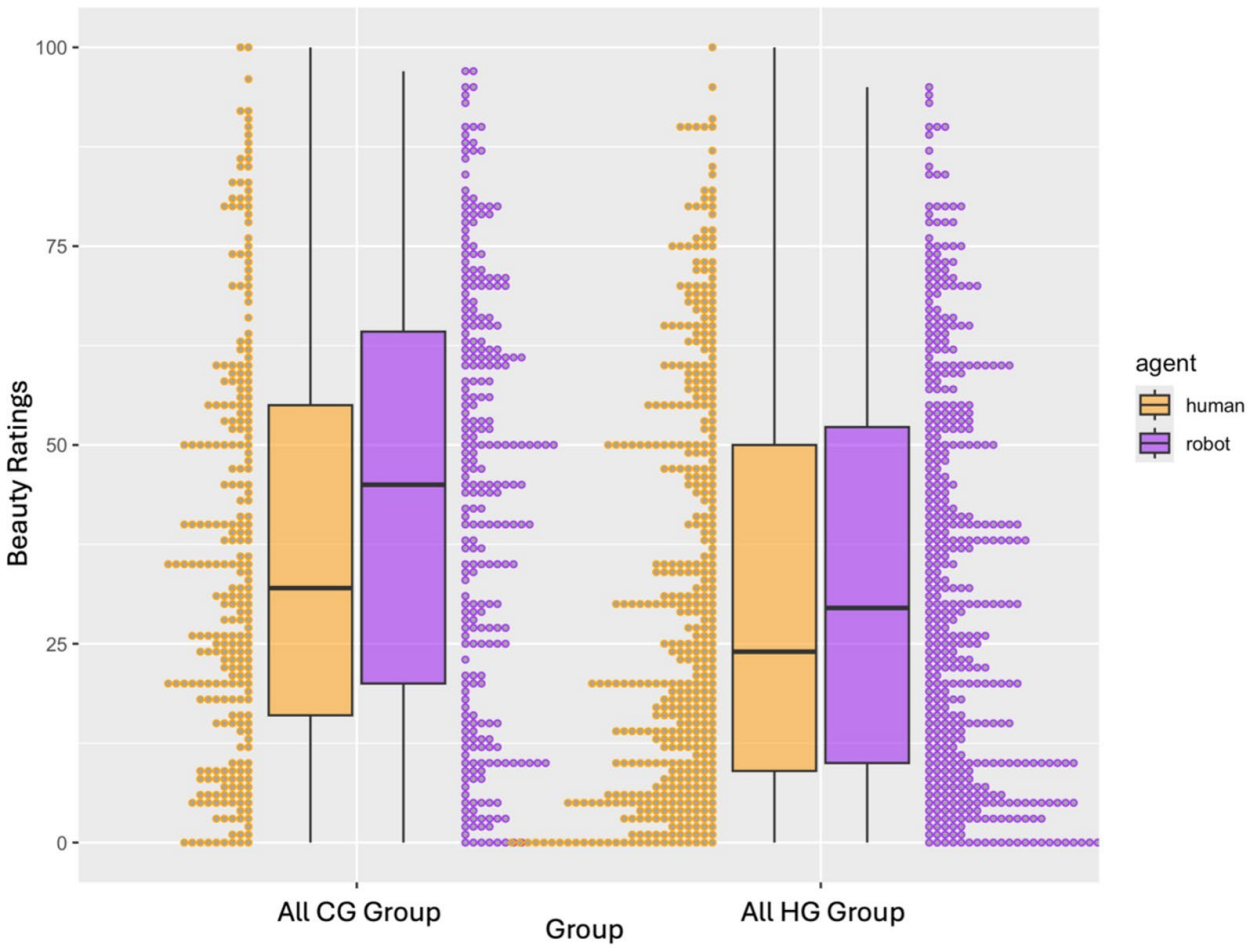
Exp 2. Interaction between agent (robot, human) and group (all CG, all HG) for beauty ratings for Experiment 2. Robot agent was rated more beautiful as opposed to human agent when the choreographies were believed to be computer generated.

*Post hoc* tests revealed that robot agent was rated as more beautiful than human agent but only in the “all CG” group which saw all original computer-generated choreographies (estimate = 4.29, SE = 1.26, *p* = 0.001, 95% CI [1.81, 6.78]), but this difference although in the same direction was not statistically significant in the “all HG” group (estimate = 0.418, SE = 1.06, *p* = 0.695, 95% CI [−1.70, 2.54]; see Figure 5 Exp 2).

Choreographies believed to be human-generated were rated as more beautiful than choreographies believed to be computer-generated in the “all CG” group (estimate = −3.685, SE = 1.65, *p* = 0.026, 95% CI [−6.93, −0.44]), but this difference was not significant in the “all HG” group (estimate = −0.46, SE = 0.87, *p* = 0.597, 95% CI [−2.17, 1.25]; see Figure 4).

##### Liking

For the simple model, a main effect of belief about the source of choreography (*p* < 0.001), group (*p* < 0.001), and the interaction between belief about source of choreography and group (*p* = 0.047) predicted liking ratings. *Post hoc* tests suggested that choreographies believed to be human generated were liked more than those believed to be computer generated but more so in “all CG” group (*p* = 0.001) and not in the “all HG” group (*p* = 0.099). However, this interaction did not persist in the full model.

For the full model, familiarity (*p* = 0.012), complexity, evocativeness, and technical complexity (ps < 0.001) positively predicted liking ratings. Difficulty of reproducing the choreography and self-reported technological expertise negatively predicted liking ratings (ps < =0.001). The main effect of agent and group predicted liking ratings such that the robot agent was liked more than the human agent (*p* = 0.007), and the “all CG” showed higher ratings of liking overall than the “all HG” group (*p* < 0.001). No other main effects or interactions were significant (see Supplementary Tables S17, S18).

##### Watch again

For the simple model, the main effect of the belief about the source of choreography (*p* = 0.001), and the interaction between choreography and group (*p* = 0.001) predicted watch again ratings. *Post hoc* tests suggested that choreographies believed to be computer generated were more likely to be watched again compared to those believed to be human generated, but only in the “all CG” group (*p* < 0.001) but not in the “all HG” group (*p* = 0.422).

This interaction persisted in the full model. For the full model, dance expertise (*p* = 0.006), familiarity, evocativeness, complexity, and technical complexity positively predicted how likely participants were to watch the video again (all ps < 0.001). The interaction between belief about the source of choreography and group predicted watch-again ratings (*p* = 0.038) in the same direction as the simple model, but pairwise comparisons did not pass our threshold of statistical significance in *post hoc* analyses (ps > 0.05; see Figure 4 from Exp 2; see Supplementary Tables S19, S20).

##### Smoothness

For the simple model, the main effect of group (*p* = 0.008), and the two-way interaction between belief about the source of choreography and group (*p* = 0.041) predicted smoothness ratings, although *post hoc* pair-wise comparisons evaluating this interaction did not pass our threshold for statistical comparison (ps > 0.05).

For the full model, familiarity, evocativeness, and technical complexity positively predicted smoothness (all ps < 0.001). Less negative/dystopian attitudes toward AI predicted higher smoothness ratings (*p* < 0.001), and age negatively predicted smoothness ratings. A main effect of belief of source of choreography (*p* = 0.003) and the two-way interaction between choreography and group (*p* = 0.005) also predicted smoothness ratings. No other main effects or interactions were statistically significant. *Post hoc* tests revealed that choreographies believed to be computer generated were rated as smoother than choreographies believed to be human generated but only for the “all CG” group (*p* = 0.001), and not for the “all HG” group (*p* = 0.844; see Figure 4 Exp 2; see Supplementary Tables S21, S22).

##### Enjoyability

For the simple model, the main effect of belief about source of choreography (*p* < 0.001), group (*p* < 0.001), and the interaction between source of choreography and group (*p* = 0.020) predicted ratings of enjoyability. *Post hoc* tests revealed that human generated choreographies were enjoyed more than choreographies believed to be computer generated in the “all CG” group (*p* = 0.0002) but only marginally so in the “all HG” group (*p* = 0.057; see Figure 4 Exp 2).

For the full model, familiarity, complexity, evocativeness, and technical complexity (all ps < 0.001) positively predicted ratings of enjoyability. Difficulty of reproducing the dance choreography (*p* = 0.012) and technological expertise (*p* < 0.001) negatively predicted ratings of enjoyability. A main effect of agent (*p* = 0.012) and a main effect of group (*p* = 0.001) predicted ratings of enjoyability such that a robot agent was found to be more enjoyable than a human agent, and ratings of enjoyability were overall higher for the “all CG” group compared to the “all HG” group.

**Figure 6:**
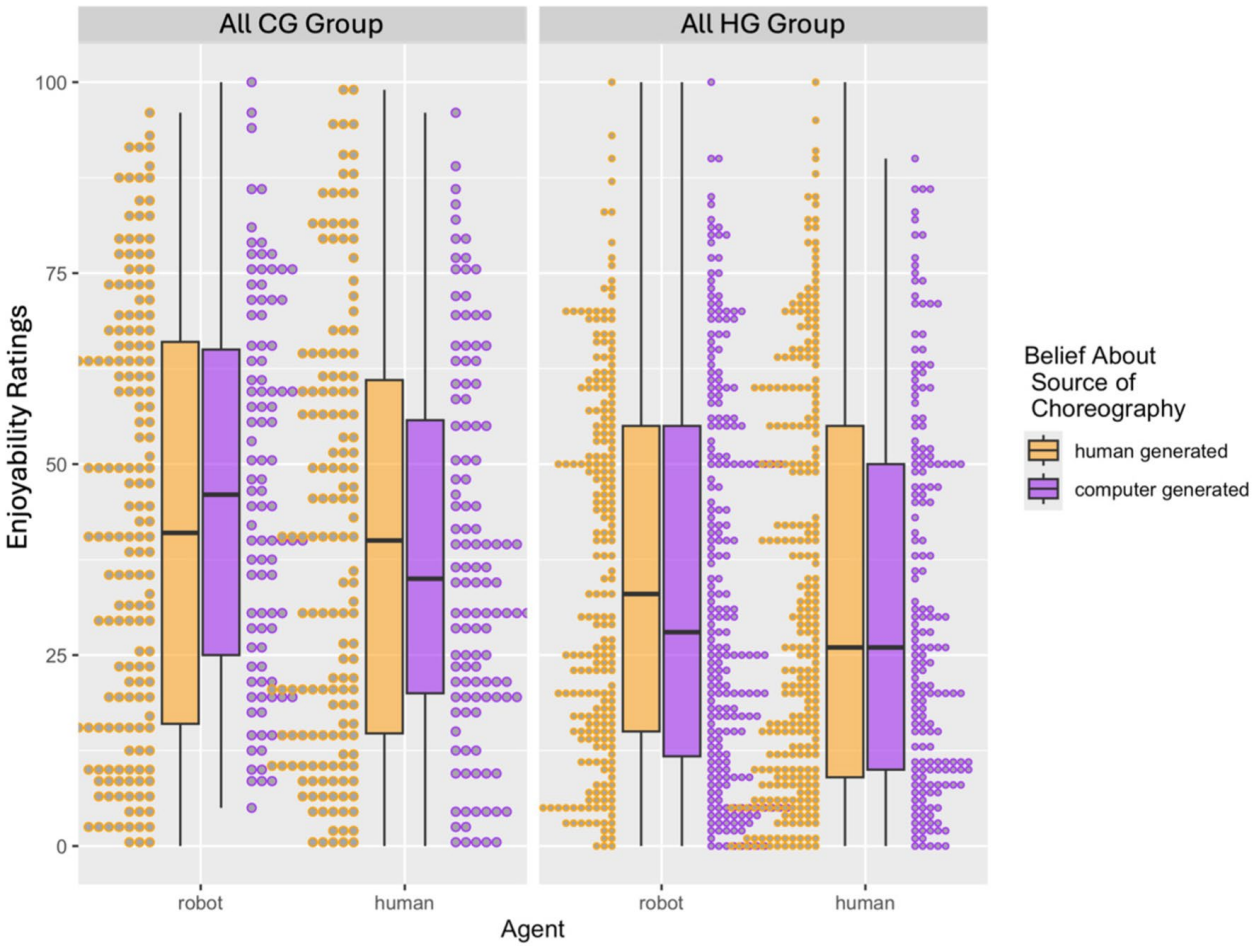
Exp 2. Three-way interaction between agent (robot, human), belief about source of choreography (human-generated, computer-generated), and group (all CG, all HG) for enjoyability ratings for Experiment 2. Enjoyability ratings were higher when the source of choreography was believed to be human generated.

The three-way interaction between agent, belief about source of choreography, and group was marginally significant (*p* = 0.086), to further evaluate the three way interaction, we tested the two-way interaction between belief about source of choreography and agent separately for the “all CG” group and “all HG” group. Separate models suggested that the interaction between agent and belief about source of choreography was marginally significant (*p* = 0.085) and the main effect of agent was significant (*p* = 0.005) for the “all CG” group such that the robot agent was rated as more enjoyable than the human agent when participants believed the choreography was human-generated (*p* = 0.004) compared to when they believed the choreography was computer generated (*p* = 0.234). For the “all HG” group, only the main effect of belief about the source of choreography predicted enjoyability ratings. The three-way interaction between agent, belief about source of choreography, and group was marginally significant (*p* = 0.086), to further evaluate the three-way interaction, we tested the two-way interaction between belief about source of choreography and agent separately for the “all CG” group and “all HG” group. Separate analyses suggested that the interaction between agent and belief about source of choreography was marginally significant (*p* = 0.085) and the main effect of agent was significant (*p* = 0.005) for the “all CG” group such that when the agent was a robot, choreographies believed to be computer generated were rated more enjoyable than choreographies believed to be human generated, and the opposite pattern was found for when the agent was a robot, although pairwise comparisons were not significant at our statistical threshold. For the “all HG” group, only the main effect of belief about the source of choreography was significant such that choreographies believed to be human-generated were rated higher on enjoyability than choreographies believed to be computer-generated (*p* = 0.021; see Figure 6 Exp 2; see Supplementary Tables S23–S26).

## Discussion

The current study investigated stimulus and knowledge cues to human animacy and their link to aesthetic appreciation of dance. Across two experiments, we explored whether the source of choreography (human-generated, computer-generated), agent (human avatar, robot avatar), and belief about the source of movement (whether created with computer animation, or human motion capture), and belief about the source of choreography (choreographies believed to be human-generated, and choreographies believed to be computer-generated) influenced ratings of how much participants liked dance videos, how beautiful they found them, how smooth they thought the videos were, the likelihood of watching the videos again, and how enjoyable they found the videos. We also tested how accurately participants were able to identify the source of choreography.

### Categorisation of human- and computer-generated dance choreographies

Results from Experiment 1 suggest that participants were not able to accurately identify whether choreographies were generated by a human or a computer. Our findings are in line with recent work that suggests accuracy is generally low when identifying whether art is made by a human or by artificial intelligence (AI; e.g., Gangadharbatla, 2022; Köbis and Mossink, 2021), but stands in contrast to other, older studies that show more accurate categorisation (e.g., Moffat and Kelly, 2006). As noted in the introduction, AI is currently progressing at a fast pace, and it is possible that as AI becomes more advanced, people are unable to distinguish between human art from computer-generated art, especially when AI is trained to imitate human art. This could also explain why we did not find a bias against computer-generated choreographies in Experiment 1, in line with previous findings from our lab (Darda and Cross, 2023).

### Aesthetic judgments are influenced by stimulus (agent) and knowledge (belief about source of movement) cues

For aesthetic ratings of beauty, liking, likelihood of watching the video again, and enjoyability, we found an interaction between agent and belief about source of movement, such that the robot avatar had higher ratings than the human avatar when participants believed the source of movement was computer animation. These findings are in contrast to evidence from social cognition that show more engagement of brain regions associated with the perception of movement for more humanlike agents, as well as more engagement of brain regions associated with social perception for movements believed to have originated from a human (rather than computer) source (e.g., Cross et al., 2016; Press, 2011; Gowen and Poliakoff, 2012). One explanation for our findings is that when the source of movement was believed to be computer animation, a robotic agent might fall into the same category (i.e., artificial movement, artificial agent) and therefore be perceived as more aesthetic by the viewers. However, previous work that has evaluated stimulus and knowledge cues to human animacy did not control for other variables that have been known to influence aesthetic appreciation. Here, we find that this two-way interaction does not hold when accounting for the influence of age, attitudes toward AI, dance and technological expertise, as well as familiarity with the choreography, technical competency of the dance, evocativeness, complexity, and difficulty of reproducing the dance by the viewer. This finding suggests that any differences between agents and belief about the source of movement might be explained by characteristics of the viewer or the dance. An interesting avenue for future research therefore would be to explore which factors or characteristics (or combination thereof) of the viewer and the dance have the most impact on aesthetic evaluations (Filippo et al., 2023).

Furthermore, in contrast to previous work (Cross et al., 2016) and when accounting for control variables, we found that when the source of movement was believed to be computer animation, videos were rated as smoother than when the source of movement was believed to be human motion capture. One difference between the current study and that reported by Cross et al. (2016) was that the videos used in the latter study were simple bimanual actions such as opening a box, sorting beads, and so on. In the current study, we used complex Bharatanatyam dance movements. It is possible that dance movements were evaluated differently than every-day (object), goal-directed actions. It is also possible that since technology has evolved at such a fast pace, and people are more exposed to and have greater overall familiarity with computer animation in recent times (Zhao et al., 2022), the participants in the present study had a higher confidence in the video’s quality.

In a similar vein, we also found that robot avatars were more likely to be watched again than human avatars. One explanation for this could be the impact of novelty. Findings from the domain of empirical aesthetics suggest that novelty is an important feature of aesthetic appreciation (e.g., Song et al., 2021). It is possible that the robot agent looked more novel than the human agent, and therefore generated curiosity in the mind of the viewer, making them more likely to watch the video again. An alternative but complementary possibility is that viewers might expect human dancers (or in this case the human agent) to dance with more finesse than a robot agent, and may therefore be more disappointed in the human agent’s performance, making it less likely for them to watch the video again. The disappointment could be a function of loss of detailed movements due to the conversion of the original videos into dances performed by a robot and human avatar. Indeed, dances such as Bharatanatyam include very fine hand gestures and movements (*mudras*) that add beauty to the dance movement. However, the stimuli we used in the study were not able to capture such fine movements when the original movement was converted into movements performed by human or robot avatars. Future research is needed to validate these propositions empirically.

Further, we found that in Experiment 2, the robot agent was found to be more beautiful and liked more than the human agent, irrespective of whether people believed the choreography was human-or computer-generated. This finding replicates findings from Experiment 1, and can perhaps be explained by the novelty of the robot agent and/or disappointment in the performance of the human agent (as discussed previously).

### Control variables and their impact on aesthetic judgements

In line with findings from empirical aesthetics, we found that ratings of familiarity, complexity, technical competency, and evocativeness all influenced ratings of liking, beauty, watch again, smoothness, and enjoyability (e.g., Orlandi et al., 2020; Cross et al., 2011; Darda and Cross, 2023). However, prior findings have demonstrated a “Cirque du Soleil” effect – art perceived as more difficult to make is liked more than art perceived as easier to make (Kruger et al., 2004), and dance movements judged as more difficult to reproduce are liked more than those judged as easier to reproduce (Cross et al., 2011). In our study, however, difficulty of reproducing the movement did not predict aesthetic ratings to a large extent.

Results from Experiment 2 also showed similar results for the control variables of familiarity, technical complexity, evocativeness, and complexity: these variables positively predicted aesthetic ratings. Interestingly, the difficulty of reproducing a movement negatively correlated with liking. That is, the more difficult to reproduce, the less liked the video, which again is in contrast to previous findings of the “Cirque du Soleil” effect (Cross et al., 2011; Orlandi et al., 2020). Indeed, some conceptual differences exist in previous work and the current study – one, we used Bharatanatyam dance (compared to ballet, modern, or other *western* dances which are more common in empirical investigations of dance aesthetics); two, we used both human and robot agents to perform the movement whereas previous studies that found a positive correlation between difficulty of reproducing a movement and aesthetic judgments have used human agents. It is possible that these differences are an explanation of our contrasting findings, but future empirical investigations are necessary to explore this claim.

### Preference for human-generated choreography?

Choreography believed to be human-generated was found to be more beautiful than computer-generated choreography, irrespective of the agent who performed the movement, a finding consistent with previous work that shows an anthropocentric bias in creative productions (Darda and Cross, 2023). Thus, in the “all CG” stimulus and knowledge cues to human animacy worked independent of each other to influence beauty ratings.

It is of note that the difference between choreographies believed to be human-generated and those believed to be computer-generated was higher in the “all CG” group than the “all HG” group. In other words, in the group that saw all computer-generated choreographies but believed some choreographies were human-generated, the bias toward choreographies believed to be human-generated was higher compared to the group which saw all human-generated choreographies but believed some choreographies were computer-generated. One possible explanation for this finding could be that a pro-human bias works more strongly than an anti-computer bias. That is, when participants believe computer-generated choreographies to be human, they show a higher bias toward human-generated choreographies than when they believe human-generated choreographies to be computer-generated. Since this was a between-groups design, future investigations can look at manipulating the actual source of choreography, and the belief about the source of choreography within the same group of participants.

Findings were in the opposite direction for the variables of smoothness and watch-again. That is, in the “all CG” group (but not the “all HG” group), people were more likely to watch videos again, and perceived them as smoother, if they believed the choreography was computer-generated than when they believed it to be human-generated. Similar to the preference for robots and computer animation videos found to be smoother in Experiment 1, it is possible that choreographies believed to be computer-generated were thought to be more novel or *different* than those believed to be human-generated, and therefore prompted viewers to see them again.

### Effect of dance and technological expertise, as well as positive and negative attitudes toward AI

Technological expertise showed a negative correlation with ratings of beauty and liking. We measured technological expertise by asking people their expertise or experience with machines and computers. A potential explanation for this finding could be that people higher on self-reported technological expertise found our videos technologically not as appealing as those they might be exposed to in media such as films or video games. Indeed, the quality of avatars created in video games is higher than the quality of videos we produced by manipulating our original dance videos shot on a green screen background. Thus, those with higher exposure to such videos, and higher expertise with perceiving and generating such videos might have found our videos less appealing, explaining the negative correlation. Dance expertise positively predicted how likely participants were to watch the video again, which is in line with previous work that suggests dance experience influences aesthetic judgments (e.g., Darda and Cross, 2023; Kirsch et al., 2015; Orgs et al., 2018).

People who showed lower dystopian views toward AI also rated videos as smoother, and age negatively predicted ratings of smoothness. These findings are in line with previous work that has found that positive attitudes toward AI increases trust in the technology and purchase intention (e.g., Liefooghe et al., 2023; Balas and Pacella, 2017). Thus, in this study, people with less negative or dystopian attitudes toward AI found the videos we created smoother as they trusted the technology with which the videos were created. In a similar vein, previous findings suggest that older people were less familiar with, and tended to have less trust in AI and technology (Gillath et al., 2021). They were also less likely to use technologies and machines in general (Heezen, 2023), and this can potentially explain the negative relationship between age and smoothness.

### Agent congruence for enjoyability

Finally, when it came to enjoyability, people who watched all human-generated choreographies but believed some were computer-generated enjoyed human-generated choreographies more than the choreographies they believed were computer-generated, irrespective of whether it was a human agent or robot agent. This finding is in line with previous work that suggests a bias against computer-generated creative productions (Darda and Cross, 2023). Here, the knowledge cue to human animacy was stronger than the stimulus cue. However, in participants who saw all computer-generated choreographies and believed some of them were human-generated, stimulus and knowledge cues interacted with each other. That is, participants enjoyed choreographies they believed were human-generated more than computer-generated choreographies when they were performed by a human agent, and they enjoyed choreographies they believed were computer-generated more than human-generated choreographies when they were performed by a robot agent.

In other words, participants preferred human agents performing movement they believed was created by humans, and they preferred robot agents performing movement they believed was created by a computer. Our findings can perhaps be supported by the *categorical perception hypothesis.* According to this hypothesis, we tend to perceive our world in terms of the categories we have formed (Harnad, 2017). Objects within categories are perceived more similar to each other than objects that belong in different categories. In our context, it is possible that when a human dancer performs a computer-generated choreography (different categories), a cognitive conflict may arise as to whether the agent is a human or non-human entity. Such categorical ambiguity may need higher cognitive processing, resulting in lower enjoyability (Cheetham et al., 2013; Wiese and Weis, 2020).

A related theory, the *perceptual mismatch hypothesis*, suggests that negative evaluations may be elicited due to a perceptual mismatch between human and artificial features (MacDorman et al., 2009; Kätsyri et al., 2015). This perceptual mismatch may be due to inconsistency between the human likeness of sensory cues (for example, artificial eyes on an otherwise human face or vice versa). In the current study, a perceptual mismatch may have been caused by a robot agent performing a choreography that the viewer perceives and believes to be human-generated (or vice versa) leading to lower ratings of the opposite pairing between agent and belief about the source of choreography. Future work will be needed to explore whether perceptual mismatch or categorical perception underlie the current results, and why an interaction between stimulus and knowledge cues might exist only when people watch all computer-generated choreographies (and believe some are human-generated), and not when they see all human-generated choreographies (and believe some are computer-generated).

### Limitations and implications

We acknowledge certain limitations of the current study. For instance, the stimuli we used were grayscale which could have led to visual information loss, leading to an incomplete perception by the audience as well as reduced ecological validity. As such, the generalisability of the findings may be limited. Future studies could look at using more detail-rich videos in naturalistic settings.

The current study has implications for multiple fields including but not limited to social robotics, social cognition, art and empirical aesthetics, as well as human-computer interaction design and engineering. The blurring line between AI and human creations not only makes us question what is “human” and what is “artificial” but can also lead to novel collaborations where human and non-human agents are part of the same system. An example of such a collaboration is Double Agent (Biggs et al., 2022) commissioned by the Museum of Discovery in Adelaide. In this installation, one agent interacts with people in the installation space, and the other agent interacts with a simulated human who has learned how to dance through the use of machine learning algorithms. Thus, Double Agent prompts the viewer to consider the role of agency within such complex systems, whether human, machine, or hybrid.

Understanding that novelty and agent congruence, as well as audience demographics, attitudes toward AI, and technological expertise can influence engagement can help AI engineers and roboticists design products and content more strategically to capture audience interest and enhance interactions with AI and machines. This can help make them more appealing by aligning their functions with user expectations, designing more user-friendly interfaces and experiences, particularly for older adults who may be less trusting of machines and AI. The current findings also have implications for the field of empirical aesthetics, highlighting the importance of controlling for factors that might influence the perception of AI/computer-generated artistic productions. Precise articulation of what these factors might be and how they influence the relationship between stimulus and knowledge cues to human animacy and aesthetic engagement presents an intriguing challenge for future research.

Our findings further suggest that only similarity with the agent is not a determining factor for how the agent and the agent’s performance or movement is perceived. Research in social cognition and social robotics suggests that how humanlike (“like-me”) an agent is influences how we perceive and engage with these agents (Ishiguro, 2013; Asada et al., 2001; Cross et al., 2016). However, the results of this study imply that the “like-me”-ness of agents may not be solely determined by its appearance (stimulus cue) but also pre-existing notions and belief that people hold about the movement and creative production’s origin (knowledge cues). Thus, along with creating more refined creative productions by using AI in a dancemaker’s toolkit, shaping people’s perceptions will be just as crucial for its reception and for better engagement.

Aesthetic ability is often considered as an advanced cognitive ability of humans, thus attracting many researchers to an area with growing interest – robotic dance. Through the application of AI and human-robot interaction technologies, researchers are working on the development of robotic dance in terms of interaction, imitation, coordination, and autonomy (Li et al., 2020). Our findings provide valuable insights to the design and development of robotic dance by identifying the features of a dance choreography (e.g., complexity, technical complexity, evocativeness, etc.) that influence its aesthetic engagement, along with audience characteristics (expertise, age, attitudes toward AI, etc.). This can serve as a valuable starting point for training machine learning algorithms to predict the link between input attributes and the predicted target for new choreographies (De Filippo et al., 2022).

Finally, it is possible that the preference toward robot avatars could carry potential negative consequences. The increasing preference for artificial agents can lead to dehumanisation of human-generated art by reducing opportunities for human performers, as well as a loss of cultural nuance and intentionality of human performers if standardised or algorithmically generated movements take precedence. It also raises ethical considerations about the nature of performance, such as whether the data the performance is based on is representative, as well as questions about authenticity. Yet, technological advancements offer exciting possibilities, and many artists see the potential that artificial intelligence and artificial agents offer for enhancing, supporting, and collaborating in artistic endeavours, rather than serving as replacements for humans.

## Data Availability

The original contributions presented in the study are publicly available. This data can be found here: https://osf.io/rxb89/.

## Footnotes

^1^The robot dance is an illusionary street dance where the dancer tries to mimic dancing robots. For example, the robot dance is described in David Mensours “From Abba to Zoom: A Pop Culture Encyclopedia of the Late 20th Century,” p. 403.

^2^Consider, for example, that in 2019 the number of publications on AI was at least three times higher than they were in any year between 2000 and 2014 and 12 times higher than they were in 2000. See: 2021-AI-Index-Report-_Chapter-1.pdf (stanford.edu).

^3^See https://www.newyorker.com/culture/culture-desk/the-new-poem-making-machinery.

^4^Google Trends. 2022. Google Trends. [online] Available at: <https://trends.google.de/trends/explore?date=all&q=AI%20art> [Accessed 23 July 2022].

^5^See: 2021-AI-Index-Report-_Chapter-1.pdf (stanford.edu).

^6^ https://osf.io/rxb89/
