# Supplementary material for: Dancing robots: aesthetic engagement is shaped by stimulus and knowledge cues to human animacy
Source: Front Hum Neurosci. 2024 Nov 25;18:1413066. doi: 10.3389/fnhum.2024.1413066 (PMC11625543; doi:10.3389/fnhum.2024.1413066)
Supplement: Supplementary file 1 [file Table_1.DOCX]

**Supplementary Material for**

**Dancing robots: Aesthetic engagement is shaped by stimulus and knowledge cues to human animacy**

**Experiment 1**

**Table S1**

*Summary table for sample demographics*

| **Variable** | **N** | **Mean** | **Std. Dev.** | **Min** | **Pctl. 25** | **Pctl. 75** | **Max** |
| --- | --- | --- | --- | --- | --- | --- | --- |
| Age | 62 | 33.40 | 10.30 | 19.00 | 24.25 | 38.00 | 60.00 |
| Gender | 62 |  |  |  |  |  |  |
| ... 1.Men | 33 | 53% |  |  |  |  |  |
| ... 2.Women | 27 | 44% |  |  |  |  |  |
| ... non-binary | 2 | 3% |  |  |  |  |  |
| Combination | 62 |  |  |  |  |  |  |
| ... 1 | 31 | 50% |  |  |  |  |  |
| ... 2 | 31 | 50% |  |  |  |  |  |
| Time taken to complete the survey (in mins) | 62 | 43.97 | 15.03 | 22.05 | 33.90 | 48.76 | 106.90 |
| Education (highest qualification) | 62 | 3.37 | 1.07 | 1.00 | 3.00 | 4.00 | 6.00 |
| Nationality | 62 |  |  |  |  |  |  |
| ... Belgium | 1 | 2% |  |  |  |  |  |
| ... Bulgaria | 1 | 2% |  |  |  |  |  |
| ... Italy | 1 | 2% |  |  |  |  |  |
| ... Mexico | 1 | 2% |  |  |  |  |  |
| ... Poland | 1 | 2% |  |  |  |  |  |
| ... United Kingdom | 48 | 77% |  |  |  |  |  |
| ... United States of America | 9 | 15% |  |  |  |  |  |
| Race | 62 |  |  |  |  |  |  |
| ... Asian | 9 | 15% |  |  |  |  |  |
| ... Black/African American | 2 | 3% |  |  |  |  |  |
| ... Hispanic Or Latino | 2 | 3% |  |  |  |  |  |
| ... Unknown/Did not specify/other | 1 | 2% |  |  |  |  |  |
| ... White | 48 | 77% |  |  |  |  |  |
| Expertise with Technology (Self-Reported) | 62 | 3.77 | 0.78 | 2.00 | 3.00 | 4.00 | 5.00 |
| Trained in Bharatanatyam | 62 |  |  |  |  |  |  |
| ... No | 62 | 100% |  |  |  |  |  |
| Dance Expertise (Self Reported) | 62 | 1.90 | 2.10 | 0.00 | 0.00 | 3.00 | 9.00 |
| Manipulation Check | 62 |  |  |  |  |  |  |
| ... Maybe | 12 | 19% |  |  |  |  |  |
| ... No, I did not notice the manipulation | 46 | 74% |  |  |  |  |  |
| ... Yes, I noticed the manipulation | 4 | 6% |  |  |  |  |  |

**Table S2**

*Statistical estimates of the three-way model for the Beauty variable*

|  | **beauty** | | | |
| --- | --- | --- | --- | --- |
| *Predictors* | *Estimates* | *CI* | *Statistic* | *p* |
| (Intercept) | 24.81 | 20.30 – 29.31 | 10.79 | **<0.001** |
| agent | -0.55 | -2.39 – 1.29 | -0.59 | 0.557 |
| source of choreography | -0.63 | -2.47 – 1.21 | -0.67 | 0.503 |
| belief about source of movement | 0.96 | -0.86 – 2.78 | 1.03 | 0.301 |
| agent × source of choreography | -0.87 | -4.55 – 2.81 | -0.47 | 0.642 |
| agent × belief about source of movement | 3.41 | -0.23 – 7.04 | 1.84 | 0.067 |
| source of choreography × belief about source of movement | 2.42 | -1.22 – 6.06 | 1.31 | 0.192 |
| (agent × source of choreography) × belief about source of movement | 4.27 | -3.01 – 11.55 | 1.15 | 0.250 |
| **Random Effects** | | | | |
| σ^2^ | 212.97 | | | |
| τ_00_ _sid_ | 313.89 | | | |
| τ_00_ _itemno_ | 3.59 | | | |
| ICC | 0.60 | | | |
| N _sid_ | 62 | | | |
| N _itemno_ | 32 | | | |
| Observations | 1984 | | | |
| Marginal R^2^ / Conditional R^2^ | 0.004 / 0.600 | | | |

**Table S3**

*Statistical estimates for Beauty while including the control variables*

|  | **beauty** | | | |
| --- | --- | --- | --- | --- |
| *Predictors* | *Estimates* | *CI* | *Statistic* | *p* |
| (Intercept) | 19.73 | 7.79 – 31.66 | 3.24 | **0.001** |
| agent | 0.18 | -0.90 – 1.25 | 0.32 | 0.749 |
| source of choreography | -0.25 | -1.33 – 0.83 | -0.46 | 0.648 |
| belief about source of movement | 1.13 | 0.06 – 2.21 | 2.06 | **0.039** |
| familiarity | 2.58 | 1.72 – 3.44 | 5.86 | **<0.001** |
| complexity | 3.83 | 2.62 – 5.03 | 6.21 | **<0.001** |
| evocative | 10.48 | 9.56 – 11.40 | 22.29 | **<0.001** |
| technical competency | 2.23 | 1.12 – 3.34 | 3.93 | **<0.001** |
| difficulty of reproducing the choreography | 0.98 | -0.16 – 2.11 | 1.69 | 0.091 |
| participant age | -0.16 | -2.23 – 1.90 | -0.16 | 0.876 |
| dance expertise | -0.82 | -2.88 – 1.24 | -0.78 | 0.437 |
| tech expertise | -1.76 | -3.87 – 0.36 | -1.63 | 0.103 |
| positive scale | 2.80 | -1.07 – 6.66 | 1.42 | 0.156 |
| negative scale | -1.43 | -5.48 – 2.62 | -0.69 | 0.490 |
| agent × source of choreography | 0.19 | -1.96 – 2.34 | 0.17 | 0.865 |
| agent × belief about source of movement | -0.17 | -2.34 – 1.99 | -0.16 | 0.875 |
| source of choreography × belief about source of movement | 2.31 | 0.16 – 4.46 | 2.11 | **0.035** |
| (agent × source of choreography) × belief about source of movement | -1.24 | -5.55 – 3.07 | -0.56 | 0.572 |
| **Random Effects** | | | | |
| σ^2^ | 132.61 | | | |
| τ_00_ _sid_ | 55.28 | | | |
| τ_00_ _itemno_ | 0.26 | | | |
| ICC | 0.30 | | | |
| N _sid_ | 62 | | | |
| N _itemno_ | 32 | | | |
| Observations | 1984 | | | |
| Marginal R^2^ / Conditional R^2^ | 0.606 / 0.722 | | | |

**Table S4**

*Statistical estimates for three-way model for the Liking Variable*

|  | **liking** | | | |
| --- | --- | --- | --- | --- |
| *Predictors* | *Estimates* | *CI* | *Statistic* | *p* |
| (Intercept) | 26.55 | 21.76 – 31.35 | 10.86 | **<0.001** |
| agent | -1.48 | -3.20 – 0.24 | -1.68 | 0.092 |
| source of choreography | -0.65 | -2.37 – 1.07 | -0.74 | 0.459 |
| belief about source of movement | 0.01 | -1.70 – 1.71 | 0.01 | 0.994 |
| agent × source of choreography | -0.48 | -3.92 – 2.96 | -0.27 | 0.786 |
| agent × belief about source of movement | 5.66 | 2.25 – 9.07 | 3.26 | **0.001** |
| source of choreography × belief about source of movement | 1.39 | -2.02 – 4.80 | 0.80 | 0.425 |
| (agent × source of choreography) × belief about source of movement | 9.11 | 2.30 – 15.93 | 2.62 | **0.009** |
| **Random Effects** | | | | |
| σ^2^ | 199.77 | | | |
| τ_00_ _sid_ | 358.95 | | | |
| τ_00_ _itemno_ | 2.93 | | | |
| ICC | 0.64 | | | |
| N _sid_ | 62 | | | |
| N _itemno_ | 32 | | | |
| Observations | 1984 | | | |
| Marginal R^2^ / Conditional R^2^ | 0.007 / 0.647 | | | |

**Table S5**

*Statistical estimates for Liking when choreographies are all computer animation*

|  | **liking** | | | |
| --- | --- | --- | --- | --- |
| *Predictors* | *Estimates* | *CI* | *Statistic* | *p* |
| (Intercept) | 26.57 | 21.56 – 31.57 | 10.41 | **<0.001** |
| agent | -4.28 | -6.29 – -2.27 | -4.18 | **<0.001** |
| source of choreography | -1.31 | -3.32 – 0.70 | -1.28 | 0.200 |
| agent × source of choreography | -4.97 | -8.99 – -0.95 | -2.43 | **0.015** |
| **Random Effects** | | | | |
| σ^2^ | 188.08 | | | |
| τ_00_ _sid_ | 387.62 | | | |
| τ_00_ _itemno_ | 1.24 | | | |
| ICC | 0.67 | | | |
| N _sid_ | 62 | | | |
| N _itemno_ | 17 | | | |
| Observations | 1008 | | | |
| Marginal R^2^ / Conditional R^2^ | 0.011 / 0.678 | | | |

**Table S6**

*Statistical estimates for Liking when choreographies are all human motion capture*

|  | **liking** | | | |
| --- | --- | --- | --- | --- |
| *Predictors* | *Estimates* | *CI* | *Statistic* | *p* |
| (Intercept) | 26.49 | 21.63 – 31.34 | 10.70 | **<0.001** |
| agent | 1.21 | -1.58 – 4.00 | 0.85 | 0.395 |
| source of choreography | -0.10 | -2.88 – 2.69 | -0.07 | 0.945 |
| agent × source of choreography | 3.80 | -1.78 – 9.37 | 1.34 | 0.181 |
| **Random Effects** | | | | |
| σ^2^ | 193.82 | | | |
| τ_00_ _sid_ | 348.34 | | | |
| τ_00_ _itemno_ | 4.87 | | | |
| ICC | 0.65 | | | |
| N _sid_ | 62 | | | |
| N _itemno_ | 16 | | | |
| Observations | 976 | | | |
| Marginal R^2^ / Conditional R^2^ | 0.002 / 0.646 | | | |

**Table S7**

*Statistical estimates for Liking while including the control variables*

|  | **liking** | | | |
| --- | --- | --- | --- | --- |
| *Predictors* | *Estimates* | *CI* | *Statistic* | *p* |
| (Intercept) | 19.88 | 4.34 – 35.42 | 2.51 | **0.012** |
| agent | -0.77 | -1.74 – 0.21 | -1.54 | 0.123 |
| source of choreography | -0.42 | -1.40 – 0.57 | -0.83 | 0.407 |
| belief about source of movement | 0.15 | -0.83 – 1.13 | 0.30 | 0.764 |
| familiarity | 4.16 | 3.31 – 5.00 | 9.63 | **<0.001** |
| complexity | 4.14 | 2.97 – 5.31 | 6.96 | **<0.001** |
| evocative | 9.42 | 8.52 – 10.31 | 20.57 | **<0.001** |
| technical competency | 2.79 | 1.71 – 3.86 | 5.09 | **<0.001** |
| difficulty of reproducing the choreography | -0.12 | -1.22 – 0.98 | -0.22 | 0.825 |
| participant age | -0.93 | -3.63 – 1.76 | -0.68 | 0.496 |
| dance expertise | -0.88 | -3.57 – 1.80 | -0.65 | 0.518 |
| tech expertise | -1.01 | -3.76 – 1.74 | -0.72 | 0.471 |
| positive scale | 0.90 | -4.14 – 5.94 | 0.35 | 0.727 |
| negative scale | 1.23 | -4.05 – 6.50 | 0.46 | 0.648 |
| agent × source of choreography | 0.43 | -1.52 – 2.38 | 0.43 | 0.666 |
| agent × belief about source of movement | 1.84 | -0.13 – 3.81 | 1.83 | 0.067 |
| source of choreography × belief about source of movement | 1.02 | -0.93 – 2.98 | 1.02 | 0.306 |
| (agent × source of choreography) × belief about source of movement | 3.30 | -0.62 – 7.22 | 1.65 | 0.099 |
| **Random Effects** | | | | |
| σ^2^ | 122.42 | | | |
| τ_00_ _sid_ | 97.16 | | | |
| ICC | 0.44 | | | |
| N _sid_ | 62 | | | |
| Observations | 1984 | | | |
| Marginal R^2^ / Conditional R^2^ | 0.559 / 0.754 | | | |

**Table S8**

*Statistical estimates of the three-way model for the Smoothness variable*

|  | **smooth** | | | |
| --- | --- | --- | --- | --- |
| *Predictors* | *Estimates* | *CI* | *Statistic* | *p* |
| (Intercept) | 42.39 | 37.47 – 47.30 | 16.92 | **<0.001** |
| agent | -1.33 | -3.51 – 0.85 | -1.20 | 0.232 |
| source of choreography | -1.40 | -3.58 – 0.78 | -1.26 | 0.207 |
| belief about source of movement | -1.94 | -4.09 – 0.22 | -1.76 | 0.078 |
| agent × source of choreography | -2.83 | -7.19 – 1.53 | -1.27 | 0.203 |
| agent × belief about source of movement | 3.97 | -0.34 – 8.28 | 1.81 | 0.071 |
| source of choreography × belief about source of movement | 0.27 | -4.04 – 4.58 | 0.12 | 0.902 |
| (agent × source of choreography) × belief about source of movement | 3.90 | -4.72 – 12.51 | 0.89 | 0.375 |
| **Random Effects** | | | | |
| σ^2^ | 282.43 | | | |
| τ_00_ _sid_ | 370.15 | | | |
| τ_00_ _itemno_ | 5.32 | | | |
| ICC | 0.57 | | | |
| N _sid_ | 62 | | | |
| N _itemno_ | 32 | | | |
| Observations | 1984 | | | |
| Marginal R^2^ / Conditional R^2^ | 0.005 / 0.573 | | | |

**Table S9**

*Statistical estimates for Smoothness while including control variables*

|  | **smooth** | | | |
| --- | --- | --- | --- | --- |
| *Predictors* | *Estimates* | *CI* | *Statistic* | *p* |
| (Intercept) | 24.98 | 4.43 – 45.52 | 2.38 | **0.017** |
| agent | -0.77 | -2.37 – 0.83 | -0.94 | 0.345 |
| source of choreography | -0.99 | -2.60 – 0.61 | -1.21 | 0.226 |
| belief about source of movement | -1.48 | -3.08 – 0.11 | -1.82 | 0.068 |
| familiarity | 3.07 | 1.94 – 4.21 | 5.30 | **<0.001** |
| complexity | 3.00 | 1.43 – 4.57 | 3.75 | **<0.001** |
| evocative | 5.86 | 4.65 – 7.06 | 9.53 | **<0.001** |
| technical competency | 5.81 | 4.36 – 7.25 | 7.89 | **<0.001** |
| difficulty of reproducing the choreography | 0.53 | -0.95 – 2.01 | 0.70 | 0.483 |
| participant age | -1.21 | -4.77 – 2.35 | -0.67 | 0.504 |
| dance expertise | -1.01 | -4.55 – 2.54 | -0.56 | 0.577 |
| tech expertise | -0.85 | -4.49 – 2.78 | -0.46 | 0.646 |
| positive scale | 3.75 | -2.91 – 10.40 | 1.10 | 0.270 |
| negative scale | 1.65 | -5.32 – 8.62 | 0.46 | 0.642 |
| agent × source of choreography | -1.67 | -4.87 – 1.54 | -1.02 | 0.307 |
| agent × belief about source of movement | 0.91 | -2.30 – 4.11 | 0.56 | 0.578 |
| source of choreography × belief about source of movement | -0.02 | -3.21 – 3.16 | -0.01 | 0.988 |
| (agent × source of choreography) × belief about source of movement | -0.72 | -7.10 – 5.67 | -0.22 | 0.826 |
| **Random Effects** | | | | |
| σ^2^ | 220.13 | | | |
| τ_00_ _sid_ | 169.48 | | | |
| τ_00_ _itemno_ | 1.77 | | | |
| ICC | 0.44 | | | |
| N _sid_ | 62 | | | |
| N _itemno_ | 32 | | | |
| Observations | 1984 | | | |
| Marginal R^2^ / Conditional R^2^ | 0.368 / 0.645 | | | |

**Table S10**

*Statistical estimates of the three-way model for the Watch Again variable*

|  | **watchagain** | | | |
| --- | --- | --- | --- | --- |
| *Predictors* | *Estimates* | *CI* | *Statistic* | *p* |
| (Intercept) | 19.27 | 14.83 – 23.72 | 8.50 | **<0.001** |
| agent | -1.88 | -3.37 – -0.38 | -2.47 | **0.014** |
| source of choreography | -0.78 | -2.28 – 0.71 | -1.03 | 0.305 |
| belief about source of movement | 0.22 | -1.26 – 1.71 | 0.29 | 0.770 |
| agent × source of choreography | -1.13 | -4.12 – 1.86 | -0.74 | 0.457 |
| agent × belief about source of movement | 3.47 | 0.50 – 6.44 | 2.29 | **0.022** |
| source of choreography × belief about source of movement | 0.83 | -2.14 – 3.79 | 0.55 | 0.585 |
| (agent × source of choreography) × belief about source of movement | 3.90 | -2.03 – 9.84 | 1.29 | 0.197 |
| **Random Effects** | | | | |
| σ^2^ | 172.81 | | | |
| τ_00_ _sid_ | 309.57 | | | |
| τ_00_ _itemno_ | 1.85 | | | |
| ICC | 0.64 | | | |
| N _sid_ | 62 | | | |
| N _itemno_ | 32 | | | |
| Observations | 1984 | | | |
| Marginal R^2^ / Conditional R^2^ | 0.005 / 0.645 | | | |

**Table S11**

*Statistical estimates for Watch Again variable while including the control variables*

|  | **watchagain** | | | |
| --- | --- | --- | --- | --- |
| *Predictors* | *Estimates* | *CI* | *Statistic* | *p* |
| (Intercept) | 26.13 | 8.67 – 43.59 | 2.94 | **0.003** |
| agent | -1.33 | -2.33 – -0.33 | -2.60 | **0.009** |
| source of choreography | -0.41 | -1.42 – 0.59 | -0.81 | 0.419 |
| belief about source of movement | 0.46 | -0.55 – 1.46 | 0.89 | 0.371 |
| familiarity | 2.02 | 1.15 – 2.89 | 4.55 | **<0.001** |
| complexity | 3.78 | 2.59 – 4.98 | 6.20 | **<0.001** |
| evocative | 6.92 | 6.00 – 7.85 | 14.71 | **<0.001** |
| technical competency | 1.66 | 0.56 – 2.76 | 2.95 | **0.003** |
| difficulty of reproducing the choreography | 0.71 | -0.42 – 1.84 | 1.23 | 0.217 |
| participant age | 0.21 | -2.81 – 3.24 | 0.14 | 0.889 |
| dance expertise | -0.94 | -3.95 – 2.07 | -0.61 | 0.540 |
| tech expertise | -0.00 | -3.09 – 3.09 | -0.00 | 0.999 |
| positive scale | -0.15 | -5.82 – 5.51 | -0.05 | 0.957 |
| negative scale | -2.13 | -8.05 – 3.79 | -0.71 | 0.480 |
| agent × source of choreography | -0.24 | -2.25 – 1.76 | -0.24 | 0.811 |
| agent × belief about source of movement | 0.84 | -1.18 – 2.86 | 0.82 | 0.414 |
| source of choreography × belief about source of movement | 0.78 | -1.23 – 2.78 | 0.76 | 0.446 |
| (agent × source of choreography) × belief about source of movement | -0.30 | -4.32 – 3.72 | -0.15 | 0.883 |
| **Random Effects** | | | | |
| σ^2^ | 128.66 | | | |
| τ_00_ _sid_ | 123.45 | | | |
| ICC | 0.49 | | | |
| N _sid_ | 62 | | | |
| Observations | 1984 | | | |
| Marginal R^2^ / Conditional R^2^ | 0.388 / 0.688 | | | |

**Table S12**

*Statistical estimates of the three-way model for the Enjoyability variables*

|  | **enjoyability** | | | |
| --- | --- | --- | --- | --- |
| *Predictors* | *Estimates* | *CI* | *Statistic* | *p* |
| (Intercept) | 26.20 | 21.44 – 30.96 | 10.80 | **<0.001** |
| agent | -0.85 | -2.79 – 1.09 | -0.86 | 0.389 |
| source of choreography | -0.37 | -2.31 – 1.56 | -0.38 | 0.704 |
| belief about source of movement | 0.42 | -1.49 – 2.33 | 0.43 | 0.667 |
| agent × source of choreography | -0.96 | -4.83 – 2.92 | -0.48 | 0.629 |
| agent × belief about source of movement | 5.16 | 1.34 – 8.98 | 2.65 | **0.008** |
| source of choreography × belief about source of movement | 1.94 | -1.88 – 5.76 | 1.00 | 0.320 |
| (agent × source of choreography) × belief about source of movement | 5.19 | -2.45 – 12.83 | 1.33 | 0.183 |
| **Random Effects** | | | | |
| σ^2^ | 200.08 | | | |
| τ_00_ _sid_ | 349.88 | | | |
| τ_00_ _itemno_ | 4.57 | | | |
| ICC | 0.64 | | | |
| N _sid_ | 62 | | | |
| N _itemno_ | 32 | | | |
| Observations | 1984 | | | |
| Marginal R^2^ / Conditional R^2^ | 0.005 / 0.641 | | | |

**Table S13**

*Statistical estimates for Enjoyability while including the control variables*

|  | **enjoyability** | | | |
| --- | --- | --- | --- | --- |
| *Predictors* | *Estimates* | *CI* | *Statistic* | *p* |
| (Intercept) | 19.25 | 3.68 – 34.83 | 2.42 | **0.015** |
| agent | -0.15 | -1.28 – 0.98 | -0.26 | 0.794 |
| source of choreography | -0.07 | -1.20 – 1.06 | -0.13 | 0.900 |
| belief about source of movement | 0.69 | -0.43 – 1.82 | 1.21 | 0.227 |
| familiarity | 3.45 | 2.59 – 4.31 | 7.89 | **<0.001** |
| complexity | 4.05 | 2.86 – 5.23 | 6.71 | **<0.001** |
| evocative | 9.44 | 8.53 – 10.35 | 20.37 | **<0.001** |
| technical competency | 3.06 | 1.97 – 4.15 | 5.51 | **<0.001** |
| difficulty of reproducing the choreography | -0.16 | -1.27 – 0.96 | -0.28 | 0.781 |
| participant age | -0.77 | -3.47 – 1.93 | -0.56 | 0.577 |
| dance expertise | -0.91 | -3.60 – 1.77 | -0.67 | 0.506 |
| tech expertise | -0.89 | -3.64 – 1.87 | -0.63 | 0.528 |
| positive scale | 0.86 | -4.19 – 5.90 | 0.33 | 0.739 |
| negative scale | 1.36 | -3.92 – 6.65 | 0.51 | 0.612 |
| agent × source of choreography | 0.02 | -2.23 – 2.28 | 0.02 | 0.983 |
| agent × belief about source of movement | 1.73 | -0.53 – 4.00 | 1.50 | 0.133 |
| source of choreography × belief about source of movement | 1.85 | -0.40 – 4.10 | 1.61 | 0.108 |
| (agent × source of choreography) × belief about source of movement | 0.01 | -4.50 – 4.51 | 0.00 | 0.997 |
| **Random Effects** | | | | |
| σ^2^ | 125.26 | | | |
| τ_00_ _sid_ | 97.42 | | | |
| τ_00_ _itemno_ | 0.62 | | | |
| ICC | 0.44 | | | |
| N _sid_ | 62 | | | |
| N _itemno_ | 32 | | | |
| Observations | 1984 | | | |
| Marginal R^2^ / Conditional R^2^ | 0.546 / 0.745 | | | |

**Experiment 2**

**Table S14**

*Summary statistics for sample demographics. NB. Cg = All CG group. Hg = All HG group.*

| **group** | **cg** | | | **hg** | | |
| --- | --- | --- | --- | --- | --- | --- |
| **Variable** | **N** | **Mean** | **SD** | **N** | **Mean** | **SD** |
| Age | 34 | 39.82 | 13.89 | 57 | 36.47 | 11.69 |
| Expertise with Dance (Self-Reported) | 34 | 1.59 | 2.06 | 57 | 1.95 | 2.46 |
| Expertise with Technology (Self Reported) | 34 | 3.56 | 0.75 | 57 | 3.68 | 0.74 |
| AI attitudes: positive scale | 34 | 3.50 | 0.54 | 57 | 3.37 | 0.67 |
| AI attitudes: negative scale | 34 | 3.09 | 0.73 | 57 | 3.26 | 0.83 |

**Table S15**

*Statistical estimates of the three-way model for Beauty*

|  | **beauty** | | | |
| --- | --- | --- | --- | --- |
| *Predictors* | *Estimates* | *CI* | *Statistic* | *p* |
| (Intercept) | 35.65 | 31.11 – 40.20 | 15.39 | **<0.001** |
| agent | -2.92 | -5.80 – -0.03 | -1.98 | **0.048** |
| source of choreography | 8.03 | 5.28 – 10.79 | 5.72 | **<0.001** |
| group | -7.24 | -9.86 – -4.63 | -5.43 | **<0.001** |
| agent × source of choreography | -0.23 | -4.57 – 4.11 | -0.10 | 0.918 |
| agent × group | 0.03 | -5.01 – 5.07 | 0.01 | 0.992 |
| source of choreography × group | -9.05 | -14.61 – -3.49 | -3.19 | **0.001** |
| (agent × source of choreography) × group | -0.52 | -9.22 – 8.18 | -0.12 | 0.907 |
| **Random Effects** | | | | |
| σ^2^ | 392.99 | | | |
| τ_00_ _sid_ | 266.94 | | | |
| τ_00_ _itemno_ | 13.23 | | | |
| ICC | 0.42 | | | |
| N _sid_ | 64 | | | |
| N _itemno_ | 16 | | | |
| Observations | 1456 | | | |
| Marginal R^2^ / Conditional R^2^ | 0.048 / 0.444 | | | |

**Table S16**

*Statistical estimates for Beauty while including Control Variables*

|  | **beauty** | | | |
| --- | --- | --- | --- | --- |
| *Predictors* | *Estimates* | *CI* | *Statistic* | *p* |
| (Intercept) | 35.48 | 33.58 – 37.37 | 36.70 | **<0.001** |
| agent | -2.36 | -3.95 – -0.76 | -2.89 | **0.004** |
| source of choreography | 2.07 | 0.27 – 3.88 | 2.25 | **0.025** |
| group | -3.67 | -5.42 – -1.93 | -4.13 | **<0.001** |
| familiarity | 1.82 | 0.88 – 2.76 | 3.78 | **<0.001** |
| complexity | 3.38 | 1.97 – 4.79 | 4.71 | **<0.001** |
| evocative | 14.51 | 13.42 – 15.60 | 26.09 | **<0.001** |
| technical competency | 5.69 | 4.38 – 7.00 | 8.53 | **<0.001** |
| difficulty of reproducing the choreography | -1.20 | -2.53 – 0.13 | -1.77 | 0.076 |
| positive scale | 1.03 | -0.17 – 2.23 | 1.68 | 0.092 |
| negative scale | 0.24 | -0.97 – 1.44 | 0.38 | 0.701 |
| participant age | 0.20 | -1.05 – 1.45 | 0.31 | 0.757 |
| dance expertise | -0.02 | -1.11 – 1.07 | -0.04 | 0.966 |
| tech expertise | -2.38 | -3.49 – -1.27 | -4.22 | **<0.001** |
| agent × source of choreography | -1.84 | -4.59 – 0.91 | -1.31 | 0.190 |
| agent × group | 3.87 | 0.88 – 6.87 | 2.54 | **0.011** |
| source of choreography × group | -3.22 | -6.84 – 0.40 | -1.75 | 0.081 |
| (agent × source of choreography) × group | -1.00 | -6.51 – 4.52 | -0.35 | 0.724 |
| **Random Effects** | | | | |
| σ^2^ | 158.98 | | | |
| τ_00_ _sid_ | 43.93 | | | |
| τ_00_ _itemno_ | 1.55 | | | |
| ICC | 0.22 | | | |
| N _sid_ | 64 | | | |
| N _itemno_ | 16 | | | |
| Observations | 1456 | | | |
| Marginal R^2^ / Conditional R^2^ | 0.700 / 0.766 | | | |

**Table S17**

*Statistical estimates of the three-way model for Liking*

|  | **liking** | | | |
| --- | --- | --- | --- | --- |
| *Predictors* | *Estimates* | *CI* | *Statistic* | *p* |
| (Intercept) | 37.33 | 32.84 – 41.82 | 16.30 | **<0.001** |
| agent | -2.07 | -4.98 – 0.83 | -1.40 | 0.162 |
| source of choreography | 5.08 | 2.32 – 7.84 | 3.61 | **<0.001** |
| group | -7.21 | -9.83 – -4.59 | -5.40 | **<0.001** |
| agent × source of choreography | 3.04 | -1.31 – 7.39 | 1.37 | 0.170 |
| agent × group | -2.34 | -7.40 – 2.73 | -0.91 | 0.365 |
| source of choreography × group | -5.64 | -11.21 – -0.08 | -1.99 | **0.047** |
| (agent × source of choreography) × group | -3.02 | -11.74 – 5.70 | -0.68 | 0.497 |
| **Random Effects** | | | | |
| σ^2^ | 394.67 | | | |
| τ_00_ _sid_ | 255.87 | | | |
| τ_00_ _itemno_ | 13.99 | | | |
| ICC | 0.41 | | | |
| N _sid_ | 64 | | | |
| N _itemno_ | 16 | | | |
| Observations | 1456 | | | |
| Marginal R^2^ / Conditional R^2^ | 0.032 / 0.425 | | | |

**Table S18**

*Statistical estimates for Liking while including control variables*

|  | **liking** | | | |
| --- | --- | --- | --- | --- |
| *Predictors* | *Estimates* | *CI* | *Statistic* | *p* |
| (Intercept) | 37.08 | 35.38 – 38.77 | 42.88 | **<0.001** |
| agent | -1.95 | -3.36 – -0.54 | -2.71 | **0.007** |
| source of choreography | -1.14 | -2.94 – 0.65 | -1.25 | 0.212 |
| group | -3.26 | -5.01 – -1.51 | -3.65 | **<0.001** |
| familiarity | 1.22 | 0.27 – 2.16 | 2.52 | **0.012** |
| complexity | 3.27 | 1.85 – 4.69 | 4.51 | **<0.001** |
| evocative | 13.43 | 12.33 – 14.53 | 23.97 | **<0.001** |
| technical competency | 8.28 | 6.96 – 9.60 | 12.32 | **<0.001** |
| difficulty of reproducing the choreography | -2.24 | -3.58 – -0.90 | -3.28 | **0.001** |
| positive scale c | 0.18 | -1.00 – 1.37 | 0.30 | 0.761 |
| negative scale c | 0.56 | -0.63 – 1.74 | 0.92 | 0.356 |
| participant age | -0.03 | -1.26 – 1.20 | -0.05 | 0.959 |
| dance expertise | -0.91 | -2.00 – 0.17 | -1.66 | 0.097 |
| tech expertise | -2.32 | -3.41 – -1.22 | -4.15 | **<0.001** |
| agent × source of choreography | 1.28 | -1.49 – 4.05 | 0.90 | 0.366 |
| agent × group | 2.34 | -0.45 – 5.14 | 1.64 | 0.101 |
| source of choreography × group | 0.42 | -3.18 – 4.02 | 0.23 | 0.821 |
| (agent × source of choreography) × group | -3.10 | -8.66 – 2.46 | -1.09 | 0.274 |
| **Random Effects** | | | | |
| σ^2^ | 163.19 | | | |
| τ_00_ _sid_ | 37.56 | | | |
| τ_00_ _itemno_ | 0.12 | | | |
| ICC | 0.19 | | | |
| N _sid_ | 64 | | | |
| N _itemno_ | 16 | | | |
| Observations | 1456 | | | |
| Marginal R^2^ / Conditional R^2^ | 0.696 / 0.753 | | | |

**Table S19**

*Statistical estimates of the three-way model for the Watch Again variable*

|  | **watchagain** | | | |
| --- | --- | --- | --- | --- |
| *Predictors* | *Estimates* | *CI* | *Statistic* | *p* |
| (Intercept) | 27.49 | 22.65 – 32.34 | 11.13 | **<0.001** |
| agent | -1.00 | -3.58 – 1.57 | -0.77 | 0.444 |
| source of choreography | 5.56 | 2.86 – 8.25 | 4.05 | **<0.001** |
| group | -2.04 | -4.61 – 0.52 | -1.56 | 0.118 |
| agent × source of choreography | 1.00 | -3.22 – 5.22 | 0.46 | 0.643 |
| agent × group | -0.33 | -5.04 – 4.37 | -0.14 | 0.889 |
| source of choreography × group | -8.98 | -14.41 – -3.55 | -3.24 | **0.001** |
| (agent × source of choreography) × group | -1.30 | -9.76 – 7.16 | -0.30 | 0.763 |
| **Random Effects** | | | | |
| σ^2^ | 373.35 | | | |
| τ_00_ _sid_ | 344.77 | | | |
| τ_00_ _itemno_ | 5.76 | | | |
| ICC | 0.48 | | | |
| N _sid_ | 64 | | | |
| N _itemno_ | 16 | | | |
| Observations | 1456 | | | |
| Marginal R^2^ / Conditional R^2^ | 0.016 / 0.492 | | | |

**Table S20**

*Statistical estimates for Watch Again while including control variables*

|  | **watchagain** | | | |
| --- | --- | --- | --- | --- |
| *Predictors* | *Estimates* | *CI* | *Statistic* | *p* |
| (Intercept) | 27.51 | 24.94 – 30.08 | 20.99 | **<0.001** |
| agent | -0.18 | -1.68 – 1.32 | -0.23 | 0.816 |
| source of choreography | 0.86 | -1.16 – 2.88 | 0.84 | 0.403 |
| group | 0.24 | -1.72 – 2.21 | 0.24 | 0.808 |
| familiarity | 3.06 | 2.01 – 4.11 | 5.72 | **<0.001** |
| complexity | 3.69 | 2.14 – 5.23 | 4.68 | **<0.001** |
| evocative | 11.79 | 10.59 – 13.00 | 19.16 | **<0.001** |
| technical competency | 3.09 | 1.65 – 4.54 | 4.20 | **<0.001** |
| difficulty of reproducing the choreography | 0.89 | -0.57 – 2.36 | 1.20 | 0.232 |
| positive scale | -0.40 | -1.80 – 0.99 | -0.57 | 0.570 |
| negative scale | 0.48 | -0.96 – 1.91 | 0.65 | 0.517 |
| participant age | 1.02 | -0.48 – 2.52 | 1.34 | 0.180 |
| dance expertise | 1.77 | 0.50 – 3.03 | 2.74 | **0.006** |
| tech expertise | -1.41 | -2.70 – -0.11 | -2.14 | **0.033** |
| agent × source of choreography | -0.07 | -3.06 – 2.93 | -0.04 | 0.966 |
| agent × group | 2.13 | -0.86 – 5.12 | 1.40 | 0.163 |
| source of choreography × group | -4.29 | -8.35 – -0.24 | -2.08 | **0.038** |
| (agent × source of choreography) × group | -1.99 | -8.00 – 4.02 | -0.65 | 0.516 |
| **Random Effects** | | | | |
| σ^2^ | 190.69 | | | |
| τ_00_ _sid_ | 98.06 | | | |
| ICC | 0.34 | | | |
| N _sid_ | 64 | | | |
| Observations | 1456 | | | |
| Marginal R^2^ / Conditional R^2^ | 0.564 / 0.712 | | | |

**Table S21**

*Statistical estimates of the three-way model for the Smoothness variable*

|  | **smooth** | | | |
| --- | --- | --- | --- | --- |
| *Predictors* | *Estimates* | *CI* | *Statistic* | *p* |
| (Intercept) | 46.11 | 41.52 – 50.70 | 19.69 | **<0.001** |
| agent | -0.56 | -3.42 – 2.30 | -0.38 | 0.702 |
| source of choreography | -1.03 | -3.83 – 1.76 | -0.72 | 0.469 |
| group | -3.60 | -6.25 – -0.94 | -2.66 | **0.008** |
| agent × source of choreography | -0.21 | -4.61 – 4.19 | -0.09 | 0.926 |
| agent × group | -3.96 | -9.02 – 1.10 | -1.53 | 0.125 |
| source of choreography × group | 5.88 | 0.24 – 11.52 | 2.05 | **0.041** |
| (agent × source of choreography) × group | 1.07 | -7.76 – 9.89 | 0.24 | 0.812 |
| **Random Effects** | | | | |
| σ^2^ | 404.65 | | | |
| τ_00_ _sid_ | 281.57 | | | |
| τ_00_ _itemno_ | 11.19 | | | |
| ICC | 0.42 | | | |
| N _sid_ | 64 | | | |
| N _itemno_ | 16 | | | |
| Observations | 1456 | | | |
| Marginal R^2^ / Conditional R^2^ | 0.008 / 0.424 | | | |

**Table S22**

*Statistical estimates for Smoothness while including control variables*

|  | **smooth** | | | |
| --- | --- | --- | --- | --- |
| *Predictors* | *Estimates* | *CI* | *Statistic* | *p* |
| (Intercept) | 45.95 | 42.92 – 48.98 | 29.73 | **<0.001** |
| agent | -0.68 | -2.66 – 1.31 | -0.67 | 0.504 |
| source of choreography | -3.57 | -5.89 – -1.26 | -3.03 | **0.003** |
| group | -1.52 | -3.75 – 0.70 | -1.34 | 0.180 |
| familiarity | 2.62 | 1.42 – 3.81 | 4.30 | **<0.001** |
| complexity | 0.34 | -1.41 – 2.10 | 0.38 | 0.702 |
| evocative | 7.93 | 6.56 – 9.30 | 11.35 | **<0.001** |
| technical competency | 9.68 | 8.04 – 11.32 | 11.56 | **<0.001** |
| difficulty of reproducing the choreography | -0.67 | -2.33 – 0.99 | -0.79 | 0.428 |
| positive scale c | -0.72 | -2.31 – 0.87 | -0.89 | 0.374 |
| negative scale c | 5.22 | 3.58 – 6.85 | 6.27 | **<0.001** |
| participant age | -2.55 | -4.26 – -0.85 | -2.94 | **0.003** |
| dance expertise | -0.65 | -2.09 – 0.79 | -0.89 | 0.374 |
| tech expertise | 0.27 | -1.20 – 1.74 | 0.36 | 0.716 |
| agent × source of choreography | -1.20 | -4.62 – 2.21 | -0.69 | 0.489 |
| agent × group | -1.11 | -4.83 – 2.62 | -0.58 | 0.560 |
| source of choreography × group | 6.72 | 2.08 – 11.35 | 2.84 | **0.005** |
| (agent × source of choreography) × group | 1.50 | -5.35 – 8.36 | 0.43 | 0.667 |
| **Random Effects** | | | | |
| σ^2^ | 244.86 | | | |
| τ_00_ _sid_ | 127.87 | | | |
| τ_00_ _itemno_ | 2.43 | | | |
| ICC | 0.35 | | | |
| N _sid_ | 64 | | | |
| N _itemno_ | 16 | | | |
| Observations | 1456 | | | |
| Marginal R^2^ / Conditional R^2^ | 0.448 / 0.640 | | | |

**Table S23**

*Statistical estimates of the three-way model for Enjoyability variable*

|  | **enjoyability** | | | |
| --- | --- | --- | --- | --- |
| *Predictors* | *Estimates* | *CI* | *Statistic* | *p* |
| (Intercept) | 37.28 | 32.73 – 41.84 | 16.06 | **<0.001** |
| agent | -2.25 | -5.09 – 0.59 | -1.56 | 0.120 |
| source of choreography | 5.91 | 3.15 – 8.68 | 4.20 | **<0.001** |
| group | -6.96 | -9.59 – -4.33 | -5.20 | **<0.001** |
| agent × source of choreography | 3.79 | -0.56 – 8.14 | 1.71 | 0.088 |
| agent × group | -1.16 | -6.17 – 3.85 | -0.45 | 0.651 |
| source of choreography × group | -6.61 | -12.18 – -1.03 | -2.32 | **0.020** |
| (agent × source of choreography) × group | -4.93 | -13.66 – 3.79 | -1.11 | 0.268 |
| **Random Effects** | | | | |
| σ^2^ | 395.34 | | | |
| τ_00_ _sid_ | 276.57 | | | |
| τ_00_ _itemno_ | 11.15 | | | |
| ICC | 0.42 | | | |
| N _sid_ | 64 | | | |
| N _itemno_ | 16 | | | |
| Observations | 1456 | | | |
| Marginal R^2^ / Conditional R^2^ | 0.034 / 0.441 | | | |

**Table S24**

*Statistical estimates for Enjoyability while including Control variables*

|  | **enjoyability** | | | |
| --- | --- | --- | --- | --- |
| *Predictors* | *Estimates* | *CI* | *Statistic* | *p* |
| (Intercept) | 37.04 | 35.23 – 38.85 | 40.23 | **<0.001** |
| agent | -1.96 | -3.50 – -0.43 | -2.50 | **0.012** |
| source of choreography | 0.10 | -1.74 – 1.95 | 0.11 | 0.912 |
| group | -3.13 | -4.92 – -1.33 | -3.42 | **0.001** |
| familiarity | 1.63 | 0.66 – 2.60 | 3.30 | **0.001** |
| complexity | 4.22 | 2.76 – 5.67 | 5.69 | **<0.001** |
| evocative | 12.16 | 11.03 – 13.28 | 21.21 | **<0.001** |
| technical competency | 7.94 | 6.60 – 9.29 | 11.56 | **<0.001** |
| difficulty of reproducing the choreography | -1.75 | -3.12 – -0.38 | -2.50 | **0.012** |
| positive scale c | 0.05 | -1.17 – 1.27 | 0.08 | 0.934 |
| negative scale c | 1.05 | -0.17 – 2.27 | 1.68 | 0.092 |
| participant age | -0.35 | -1.61 – 0.92 | -0.54 | 0.589 |
| dance expertise | -0.62 | -1.73 – 0.49 | -1.09 | 0.274 |
| tech expertise | -2.69 | -3.81 – -1.56 | -4.69 | **<0.001** |
| agent × source of choreography | 2.15 | -0.69 – 4.99 | 1.49 | 0.138 |
| agent × group | 2.55 | -0.42 – 5.52 | 1.68 | 0.093 |
| source of choreography × group | -1.20 | -4.90 – 2.50 | -0.64 | 0.525 |
| (agent × source of choreography) × group | -4.98 | -10.67 – 0.71 | -1.72 | 0.086 |
| **Random Effects** | | | | |
| σ^2^ | 169.99 | | | |
| τ_00_ _sid_ | 41.14 | | | |
| τ_00_ _itemno_ | 0.71 | | | |
| ICC | 0.20 | | | |
| N _sid_ | 64 | | | |
| N _itemno_ | 16 | | | |
| Observations | 1456 | | | |
| Marginal R^2^ / Conditional R^2^ | 0.681 / 0.744 | | | |

**Table S25**

*Statistical estimates of Enjoyability for only Computer-generated Choreographies*

|  | **enjoyability** | | | |
| --- | --- | --- | --- | --- |
| *Predictors* | *Estimates* | *CI* | *Statistic* | *p* |
| (Intercept) | 41.77 | 35.22 – 48.31 | 12.54 | **<0.001** |
| agent | -6.00 | -10.16 – -1.85 | -2.84 | **0.005** |
| source of choreography | -1.61 | -9.86 – 6.64 | -0.38 | 0.702 |
| agent × source of choreography | 5.74 | -0.78 – 12.26 | 1.73 | 0.084 |
| **Random Effects** | | | | |
| σ^2^ | 351.55 | | | |
| τ_00_ _sid_ | 335.79 | | | |
| τ_00_ _itemno_ | 6.97 | | | |
| ICC | 0.49 | | | |
| N _sid_ | 34 | | | |
| N _itemno_ | 16 | | | |
| Observations | 544 | | | |
| Marginal R^2^ / Conditional R^2^ | 0.014 / 0.501 | | | |

**Table S26**

*Statistical estimates of Enjoyability for only Human-generated Choreographies*

|  | **enjoyability** | | | |
| --- | --- | --- | --- | --- |
| *Predictors* | *Estimates* | *CI* | *Statistic* | *p* |
| (Intercept) | 33.37 | 28.10 – 38.64 | 12.43 | **<0.001** |
| agent | -2.95 | -8.23 – 2.32 | -1.10 | 0.272 |
| source of choreography | 2.77 | 0.42 – 5.13 | 2.31 | **0.021** |
| agent × source of choreography | 1.40 | -3.31 – 6.11 | 0.58 | 0.560 |
| **Random Effects** | | | | |
| σ^2^ | 295.03 | | | |
| τ_00_ _sid_ | 307.86 | | | |
| τ_00_ _itemno_ | 23.58 | | | |
| ICC | 0.53 | | | |
| N _sid_ | 57 | | | |
| N _itemno_ | 16 | | | |
| Observations | 912 | | | |
| Marginal R^2^ / Conditional R^2^ | 0.006 / 0.532 | | | |
